# Effector function of anti-pyroglutamate-3 Aβ antibodies affects cognitive benefit, glial activation and amyloid clearance in Alzheimer’s-like mice

**DOI:** 10.1186/s13195-019-0579-8

**Published:** 2020-01-13

**Authors:** Helen Crehan, Bin Liu, Martin Kleinschmidt, Jens-Ulrich Rahfeld, Kevin X. Le, Barbara J. Caldarone, Jeffrey L. Frost, Thore Hettmann, Birgit Hutter-Paier, Brian O’Nuallain, Mi-Ae Park, Marcelo F. DiCarli, Inge Lues, Stephan Schilling, Cynthia A. Lemere

**Affiliations:** 1 Ann Romney Center for Neurologic Diseases, Brigham and Women’s Hospital, Hale BTM 9002S, 60 Fenwood Rd, Boston, MA 02115 USA; 2 Harvard Medical School, Boston, MA USA; 3 Vivoryon Therapeutics AG, Halle (Saale), Germany; 4 Department Drug Design and Target Validation, Fraunhofer Institute for Cell Therapy and Immunology, Halle (Saale), Germany; 5 Mouse Behavior Core, Harvard Medical School, Boston, MA USA; 6 QPS Austria, Grambach, Austria; 7 Department of Radiology, Brigham Women’s Hospital, Boston, MA USA

**Keywords:** Pyroglutamate-3 amyloid-β, Immunotherapy, APP_SWE_/PS1ΔE9, Phagocytosis, Microhemorrhage, microPET

## Abstract

**Background:**

Pyroglutamate-3 Aβ (pGlu-3 Aβ) is an N-terminally truncated and post-translationally modified Aβ species found in Alzheimer’s disease (AD) brain. Its increased peptide aggregation propensity and toxicity make it an attractive emerging treatment strategy for AD. We address the question of how the effector function of an anti-pGlu-3 Aβ antibody influences the efficacy of immunotherapy in mouse models with AD-like pathology.

**Methods:**

We compared two different immunoglobulin (Ig) isotypes of the same murine anti-pGlu-3 Aβ mAb (07/1 IgG1 and 07/2a IgG2a) and a general N-terminal Aβ mAb (3A1 IgG1) for their ability to clear Aβ and protect cognition in a therapeutic passive immunotherapy study in aged, plaque-rich APP_SWE_/PS1ΔE9 transgenic (Tg) mice. We also compared the ability of these antibodies and a CDC-mutant form of 07/2a (07/2a-k), engineered to avoid complement activation, to clear Aβ in an ex vivo phagocytosis assay and following treatment in APP_SL_xhQC double Tg mice, and to activate microglia using longitudinal microPET imaging with TSPO-specific ^18^F-GE180 tracer following a single bolus antibody injection in young and old Tg mice.

**Results:**

We demonstrated significant cognitive improvement, better plaque clearance, and more plaque-associated microglia in the absence of microhemorrhage in aged APP_SWE_/PS1ΔE9 Tg mice treated with 07/2a, but not 07/1 or 3A1, compared to PBS in our first in vivo study. All mAbs cleared plaques in an ex vivo assay, although 07/2a promoted the highest phagocytic activity. Compared with 07/2a, 07/2a-k showed slightly reduced affinity to Fcγ receptors CD32 and CD64, although the two antibodies had similar binding affinities to pGlu-3 Aβ. Treatment of APP_SL_xhQC mice with 07/2a and 07/2a-k mAbs in our second in vivo study showed significant plaque-lowering with both mAbs. Longitudinal ^18^F-GE180 microPET imaging revealed different temporal patterns of microglial activation for 3A1, 07/1, and 07/2a mAbs and no difference between 07/2a-k and PBS-treated Tg mice.

**Conclusion:**

Our results suggest that attenuation of behavioral deficits and clearance of amyloid is associated with strong effector function of the anti-pGlu-3 Aβ mAb in a therapeutic treatment paradigm. We present evidence that antibody engineering to reduce CDC-mediated complement binding facilitates phagocytosis of plaques without inducing neuroinflammation in vivo. Hence, the results provide implications for tailoring effector function of humanized antibodies for clinical development.

## Background

Alzheimer’s disease (AD) is the most common form of dementia, with global prevalence of this devastating disease estimated at 50 million people and predicted to triple by 2050 [1]. The pathological hallmarks of AD include amyloid-beta (Aβ) plaques and neurofibrillary tangles. Strong genetic and pathological evidence indicates the “amyloid hypothesis” as a central contributor of early AD pathogenesis [2].

On the basis of the amyloid hypothesis, removal of Aβ aggregates by immunotherapy has been suggested as a treatment option to slow or halt AD [3]. Peripherally administered anti-Aβ antibodies are able to access the CNS to reduce the extent of plaque deposition through Fc-mediated phagocytosis [4, 5]. Following the initial failures of active and passive immunotherapies after the onset of clinical symptoms, a Phase 1b study of an anti-Aβ monoclonal antibody, aducanumab, provided promising results with regard to amyloid lowering and cognitive readouts [6]. Similar to some other anti-amyloid antibodies, aducanumab treatment was accompanied by amyloid-related imaging abnormalities (ARIA), imaging abnormalities indicating mostly asymptomatic and transient side effects due to vascular edema. Biogen halted all aducanumab clinical trials in March 2019 after an interim futility analysis of half of the participants predicted that there would be no clinical benefit in patients with prodromal or mild AD (press release March 21, 2019; AD/PD 2019, Lisbon). In October 2019, Biogen announced that upon analysis of the full data set of all participants, aducanumab reached its primary endpoint of clinical benefit (CDR-SB) in a large Phase III clinical trial (EMERGE), and while not significant in another large Phase III (ENGAGE) study, post hoc analysis showed slowing of cognitive decline for those in the high dose treatment group (press release Oct 22, 2019; CTAD 2019, San Diego). Both studies showed dose- and time-dependent lowering of cerebral amyloid. Biogen announced it will seek Federal Drug Administration approval in early 2020. If approved, aducanumab would be the first-ever disease modifying treatment for mild cognitive impairment and early-stage Alzheimer’s disease.

As with aducanumab, most of the antibodies currently under clinical development are immunoglobulin G (IgG1) molecules. Immunoglobulin G (IgG) is the major class of immunoglobulins in human serum, and the Fc portion of this antibody determines the IgG isotype and, as such, mediates the effector function such as phagocytosis, antibody-dependent cellular toxicity (ADCC), and complement-dependent cytotoxicity (CDC) [7]. Human IgG1 and IgG3 usually elicit a strong effector function via Fcγ receptors [8]. IgG1 accounts for the highest abundance of immunoglobulin in the blood and has a longer half-life; thus, it has been the preferred candidate for engineering an antibody for therapeutic use [9, 10]. However, the strong effector function of IgG1 molecules might trigger neuroinflammation and ARIAs, as observed with aducanumab and other anti-amyloid antibodies, including Eli Lilly’s anti-pGlu3 Aβ antibody, donanemab which is currently in clinical trials. Other monoclonal antibodies, such as crenezumab [11], were generated on an IgG4 backbone in order to reduce ADCC and CDC functionality. However, two large Phase III crenezumab clinical trials in early AD were terminated earlier this year due to an interim analysis that suggested that treatment would not likely slow cognitive decline in patients with prodromal to mild AD. A Phase II secondary prevention study of crenezumab treatment in amyloid-positive cognitively normal subjects with the presenilin 1 E280A mutation that causes early onset familial AD is currently underway in Colombia through the Alzheimer’s Prevention Initiative. These setbacks suggest that the interplay between antibody epitope specificity and effector function requires a thorough analysis, which was one of our major goals of this current study.

In the light of these previous findings, we wanted to address the role of the IgG isotype for efficacy and potential side effects for an anti-pyroglutamate-3 (pGlu-3) Aβ antibody, 07/1 [12]. This antibody targets a truncated and N-terminally modified form Aβ40/42, which is present in the intracellular, extracellular, and vascular deposits in AD and Down syndrome (DS) brain tissue [13–16]. The formation of pGlu-3 Aβ results in altered biochemical properties such as elevated hydrophobicity, higher predisposition to aggregate and increased toxicity [17–22]. We previously reported that preventive immunization with the murine anti-pGlu-3 Aβ IgG1 07/1 mAb improved cognition and reduced plaques in young APP_SWE_/PS1ΔE9 Tg mice when administered at an early stage of plaque deposition [12]. (Note that human and mouse IgG isotypes are not directly homologous: Human IgG1 is comparable with mouse IgG2a due to similar ability to bind to Fcγ receptors and activate the complement system [23]). We also reported in a small pilot study that 07/1 reduced plaque deposition with a murine anti-pGlu3 Aβ IgG1 mAb in aged APP_SWE_/PS1ΔE9 Tg mice; however, biochemical Aβ levels were unchanged and cognition was not examined [24]. In the present study, we compared the effect of therapeutic immunization with the 07 anti-pGlu3 Aβ, of either murine IgG1 or IgG2a subtype, known as 07/1 and 07/2a respectively, and a general N-terminal Aβ mAb (3A1 IgG1) on behavior, Aβ clearance, and microglia in aged APP_SWE_/PS1ΔE9 Tg mice. In subsequent studies, a CDC mutation (K322A) was engineered into the murine 07/2a antibody to inhibit complement activation (07/2a-k mAb). The effector functions of these anti-Aβ mAbs were further examined in a double transgenic hAPPSLxhQC mouse model and an ex vivo phagocytosis assay. Lastly, the microglial activation profiles at baseline, 3 days, and 30 days after a single bolus injection of PBS or antibody were investigated by longitudinal in vivo microPET imaging using a second-generation, highly specific translocator protein (TSPO) radioligand, ^18^F-GE180 [25, 26] in both 4-month (mo)-old and 16-mo-old APP_SWE_/PS1ΔE9 Tg mice. In summary, this nonclinical study was designed to provide information regarding the effector function responsible for efficacy and potential side effects of anti-pGlu-3 Aβ mAb immunotherapy.

## Methods

### Animals

A passive immunization study was conducted in male, plaque-rich APP_SWE_/PS1ΔE9 transgenic (Tg) mice on a C57BL/6 J background starting at ~ 12 mo of age. APP_SWE_/PS1ΔE9 mice express two human genes of familial AD, the APP^K594N/M595L^ Swedish and Presenilin 1 delta E9 (PS1ΔE9) (deletion of exon 9) under a mouse prion promoter [27]. Original Tg breeders were obtained from The Jackson Laboratory (Bar Harbor, ME) and were maintained in our colony by crossing male APP_SWE_/PS1ΔE9 Tg mice with female C57BL/6 J mice. All animal protocols were approved by the Harvard Medical Area Standing Committee on Animals, and studies were performed in accordance with all state and federal regulations. The Harvard Medical School animal management program is accredited by the Association for the Assessment and Accreditation of Laboratory Animal Care International and meets all National Institutes of Health standards as demonstrated by an approved Assurance of Compliance (A3431-01) filed at the Office of Laboratory Animal Welfare.

Another amyloid mouse model, hAPPSL;hQC mice, which are double transgenic for the human APP gene containing Swedish and London mutation and human glutaminyl cyclase (QC) [19], were also used to assess the efficacy of 07/2a IgG2a and 07/2a-k IgG2a antibodies. Animals were housed in individually ventilated cages on standardized rodent bedding supplied by Rettenmaier Austria GmbH & Co.KG (Vienna, Austria). Mice were kept in the Association for Assessment and Accreditation of Laboratory Animal Care-accredited animal facility of QPS Austria GmbH (previously JSW Lifesciences, GmbH, Grambach, Austria). Animal studies conformed to the Austrian guidelines for the care and use of laboratory animals and were approved by the Styrian government, Austria.

### Antibodies for immunotherapy and the ex vivo phagocytosis assay

Multiple murine anti-pyroglutamate-3 Aβ antibodies of different IgG isotypes were used in these studies, all of which were generated and provided by Vivoryon Therapeutics AG (formerly Probiodrug AG, Halle, Germany). These include murine IgG1 (07/1) and IgG2a (07/2a) isotype versions of the same 07 monoclonal antibody targeting the N-terminally truncated and modified (by cyclization by QC) Aβ. In an effort to avoid inflammation and possibly ARIA, a CDC-mutant (K322A) version of the murine IgG2a isotype antibody (07/2a-k), which exhibits less complement activation due to its inability to bind C1q, was engineered and provided by Vivoryon Therapeutics AG. The respective IgG1 isotype control antibody was purchased from Thermo Fisher Scientific; the IgG2a isotype control was produced by Fraunhofer IZI-MWT.

In addition, the following studies included a purified β-amyloid 1–15 monoclonal antibody raised against dityrosine cross-linked human Aβ1–40, named 3A1, which was kindly provided by Dr. Brian O’Nuallain and is now commercially available from BioLegend. 3A1 does not recognize pGlu3 Aβ or APP [12].

### Immunotherapy treatment, behavioral testing, and euthanasia

A total of 80 male 12-mo-old APP_SWE_/PS1ΔE9 mice were utilized for the first in vivo study. Prior to the start of immunization, five APP_SWE_/PS1ΔE9 Tg mice (avg. 12.3 mo ± 0.08) were sacrificed as baseline controls to assess plaque burden at commencement of treatment. The remaining 75 mice were divided into four groups and received the following treatments: 250 μl sterile phosphate buffered saline (PBS) (*n* = 14; originally *n* = 15 but one removed due to incorrect genotype); avg. 12.2 mo ± 0.77), 300 μg 3A1, a general Aβ IgG1 mAb (*n* = 15; avg. 12.2 mo ± 0.8), 300 μg 07/1, a pGlu-3 Aβ IgG1 mAb (*n* = 15; avg. 12.2 mo ± 0.55) or 300 μg 07/2a, a pGlu-3 Aβ IgG2a mAb (*n* = 15; avg. 12.3 mo ± 0.35). A group of age- and gender-matched wildtype (Wt) littermates were injected with 250 μl sterile PBS (*n* = 15; avg. 12.3 mo ± 0.91) and served as behavioral controls. Mice were treated weekly with a total volume of 250 μl antibody or PBS via intraperitoneal (i.p) injection for 16 weeks (i.e., from 12 to 16 mo of age).

Behavioral testing of all mice was performed starting at ~ 15 mo of age, during which time, mice continued to be treated. Open field (to measure spontaneous locomotor activity and anxiety), contextual fear conditioning (CFC) (to measure associative learning and fear memory), and water T-maze (to assess spatial learning and memory) tests were performed as described [12]. Mice were euthanized at 16 mo of age, 1 week after the last treatment.

An additional immunotherapy study with 29 mice in total was conducted in 8-mo-old hAPPSL;hQC mice examining the in vivo effects of 07/2a IgG2a and 07/2a-k IgG2a-k. These mice were divided into three groups; PBS vehicle treated (*n* = 7), 07/2a (150 μg) (*n* = 8), 07/2a (500 μg) (*n* = 7) and 07/2a-k (500 μg) (*n* = 7) and given a weekly i.p injection for 16 weeks.

Mice were euthanized and perfused and brain and plasma were harvested prior to dosing (baseline) or after i.p injections for 16 weeks as previously described in [16] and in Additional file 1 (S1.1).

### Histology, image analysis, and ELISA quantification of Aβ and cytokines

Ten- and 20-μm-thick brain sagittal cryosections were cut with a Leica CM1850 cryostat and mounted on Colorfrost Plus slides (Fisher Scientific) or Poly-L-lysine-coated 12-mm glass coverslips (Corning) for immunohistochemistry (IHC) and an ex vivo phagocytosis assay, respectively. Neuropathological analysis of Aβ plaque burden and associated gliosis was carried out as previously described in [16] and in Additional file 1: (S1.2). Fibrillar amyloid was stained with Thioflavin S dye as previously described in [28] and in Additional file 1: (S1.2). Brain tissue without the cerebellum was homogenized. Aβ and cytokine levels in homogenates were measured by ELISA as outlined in Additional file 1: (S1.3).

### Antibody concentration measurements in plasma and brain

The concentrations of the exogenous antibodies (3A1, 07/1, and 07/2a) in the brain homogenates and the terminal plasma samples (1 week post-final injection) were measured as described [12], using streptavidin-coated 96-well plates coated in biotinylated Aβ peptide as described in Additional file 1: (S.1.4).

### Cell culture

Primary microglia (PMG) were prepared from the cortices of mouse pups at postnatal day 5 and cultured as described [29, 30]. The murine N9 immortalized microglial cell line was generated by Dr. Paola Ricciardi-Castagnoli [31] and was kindly provided to us by Dr. Joseph El Khoury (Massachusetts General Hospital) with her permission.

### Ex vivo phagocytosis assay

Aged (20-mo-old), plaque-rich APP_SWE_/PS1ΔE9 unfixed mouse brains were gently frozen in liquid nitrogen vapors, sagittally sectioned to 20 μm, and mounted on 12 mm round poly-l-lysine-coated cover slips and placed in 24-well tissue culture plates for the PMG cell assay or on 18 mm round poly-l-lysine-coated cover slips and placed in 12-well tissue culture plates for the N9 cell assay. IHC was performed to confirm tissue was plaque-rich with an abundance of pGlu3-Aβ epitope available. Serial sections were incubated with or without antibodies for 1 h at 37 °C in 5% CO_2_: anti-pGlu3 Aβ mAbs 07/1 IgG1, 07/2a IgG2a, and 07/2a-k IgG2a; anti-Aβ 3A1 IgG1; or isotype control IgG1 or isotype control IgG2a, at optimized concentrations of 10 μg/ml for the PMG cell assay or 15 μg/ml for the N9 cell assay. The assay was carried out as described by [32] and in Additional file 1 (S1.5).

### Antibody binding to murine Fcγ receptors

The binding affinities of 07/2a and 07/2a-k to the murine Fcγ receptors (CD16, CD32 and CD64) of J774 macrophages were determined with a conventional ELISA (STC Biologicals, Boston, MA). Recombinant murine CD16, CD32, or CD64 were coated onto plates and bound 07/2a and 07/2a-k antibodies were detected with an anti-mouse Fab-HRP and quantitated by colorimetric readings. Dissociation constant (K_D_) values were derived from fitted IC_50_ values using fixed bottom (0%) and top (100%) values, as well as a slope value of 1.

### In vivo PET imaging with ^18^F-GE180 PET tracer and image analysis

In vivo microPET imaging was carried out as described in Liu et al. [25] and Additional file 1 (S1.6). Briefly, 4-mo- and 16-mo-old male APP/PS1dE9 mice underwent baseline imaging at day 0 followed by an intravenous tail vein injection of 500 μg of 3A1 mAb, 07/1 mAb, 07/2a mAb, 07/2a-k mAb, or PBS the next day. Follow-up microPET scans were performed 3 days and 30 days post-injection.

### Statistics

With the exception of the behavioral data, statistical analyses were conducted with Prism 5 (GraphPad) using two-way ANOVA with Bonforroni post hoc test for PET imaging analysis or one-way ANOVA with Newman-Keuls post hoc test for all the other group analyses. Where indicated, Student’s *t* test was performed for some analyses. For behavioral data, StatView (Version 5.0) was used along with Fisher’s PLS. A *p* value of < 0.05 was considered significant, and all data are expressed as the mean ± SEM, unless otherwise stated.

## Results

### 07/2a mAb treatment significantly improved cognition

**Fig. 1:**
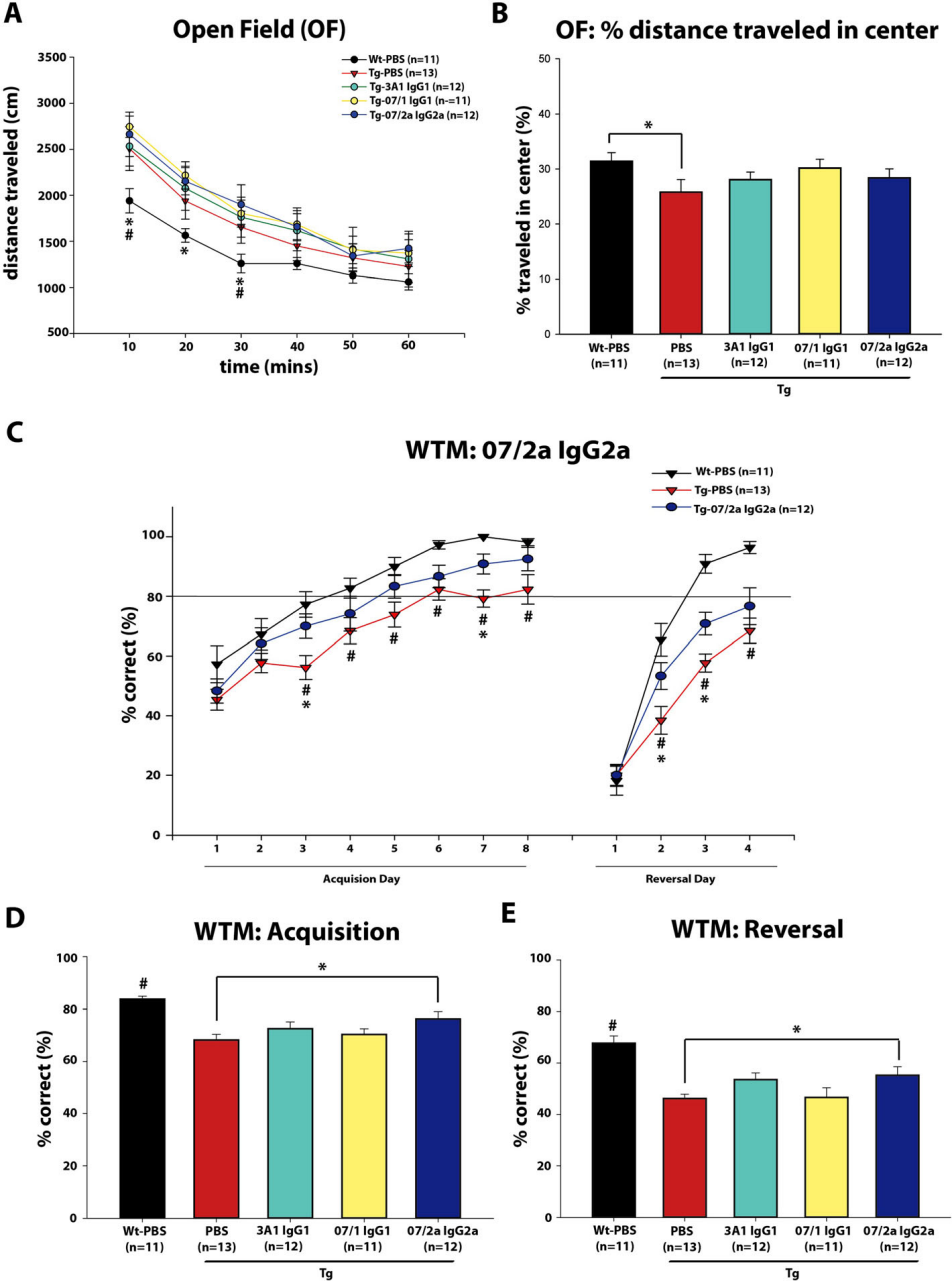
Anti-pGlu-3 Aβ IgG2a isotype mAb shows significant cognitive improvement in 15-mo-old Tg mice. No effect was observed on locomotor activity in the APP_SWE_/PS1ΔE9 Tg mice, as measured by total distance traveled in the open field (OF) (**a**). Less distance was spent in the center of the OF by PBS-treated Tg versus Wt mice (*p* < 0.05), and 07/1 treatment showed a trend (*p* < 0.0836) to reverse this (**b**). Day by day analysis of the acquisition phase of the water T-maze (WTM) shows there were fewer (*p* < 0.05) correct responses to find the platform in PBS-treated Tg versus Wt mice on days 3–8 (**c**). 07/2-treated Tg mice displayed a significant increase (*p* < 0.05) in the percent of correct responses on days 3 and 7 compared to Tg PBS-treated mice (**c**). Day by day analysis of the reversal phase of the WTM (**c**) demonstrated a reduction (*p* < 0.05) in percent of correct responses on days 2–4 by the PBS-injected Tg vs. Wt mice, indicating that the Tg mice had impaired cognitive flexibility. A significant increase in correct responses was observed in Tg mice treated with 07/2a (*p* < 0.05) on days 2 and 3 (**c**). WTM results averaged across all days confirmed that Tg mice made significantly fewer (*p* < 0.05) correct responses to find the platform compared to Wt PBS-injected mice in both the acquisition and reversal phases, which appeared to be partially rescued following 07/2a (*p* < 0.05) treatment compared to PBS Tg mice (**d**, **e**). *n* = 11–13 per group. All data are expressed as the mean ± SEM. Fisher’s PLSD: **a** *p < 0.05 Wt PBS versus Tg 3A1, Tg 07/1, and Tg 07/2a at 10 and 20 min, *p* < 0.05 Wt PBS versus Tg 07/1, Tg 07/2a at 30 min, ^#^*p* < 0.05 Wt PBS versus Tg PBS. **b**, **d**, and **e** *p < 0.05 versus Tg PBS, ^#^*p* < 0.05 Wt PBS versus all groups. **c** *p < 0.05 Tg PBS versus 07/2a, ^#^*p* < 0.05 Wt PBS versus Tg PBS

Behavioral testing was initiated at 15 mo of age, approximately 1 month prior to the mice receiving their 16th and final weekly i.p. injection. To control for non-specific effects on learning and memory, Wt littermate mice received injections of PBS. Following 13 weeks of antibody or PBS administration by i.p injection, mice were placed in an open field arena for measurement of the effects of passive immunotherapy on locomotor activity and anxiety. Total distance traveled and the percent distance traveled in the center of the field was recorded over 60 min. Antibody and PBS-treated Tg mice were compared to PBS-treated Wt control mice. As expected based on our previous studies, mAb and PBS-injected Tg mice were more active (i.e., more total distance traveled) than Wt PBS-injected mice in the first 30 min of the test session; however, no differences were observed between groups during the last 30 min of the test session (Fig. 1a). Therefore, mAb treatment did not affect locomotor activity. There was a significant decrease in percent distance traveled in the center of the open field in the Tg PBS-injected mice compared to Wt PBS controls (Fig. 1b) demonstrating a genotype-specific increase in anxiety-like behavior in APP_SWE_/PS1ΔE9 mice at 15 mo of age. There was a strong trend for an increased percent distance traveled in the center in the mice treated with 07/1 mAb (*p* = 0.54); however, this did not reach significance (Fig. 1b). No differences were observed in the 07/2a-treated Tg mice in the open field testing. CFC testing did not show any differences in fear-associated learning or memory between groups (data not shown).

A water T-maze (WTM) test was used to assess antibody treatment effects on spatial learning and memory and cognitive flexibility in the APP_SWE_/PS1ΔE9 mice. The number of correct responses to find the platform made by the PBS-injected Tg mice in the acquisition (*p* < 0.05) and reversal (*p* < 0.05) phases was significantly fewer than what was recorded by the Wt PBS-injected mice demonstrating that the APP_SWE_/PS1ΔE9 mice had learning and memory deficits at 15 mo of age (Fig. 1c–e). These deficits were attenuated in the Tg mice that were treated with 07/2a mAb, which instead showed a significant increase in the percent of correct responses in both the acquisition and reversal tests compared to the PBS-injected Tg control mice (Fig. 1d, e).

### 07/2a mAb immunotherapy reduced total Aβ plaque burden in aged APP_SWE_/PS1ΔE9 Tg mice

**Fig. 2:**
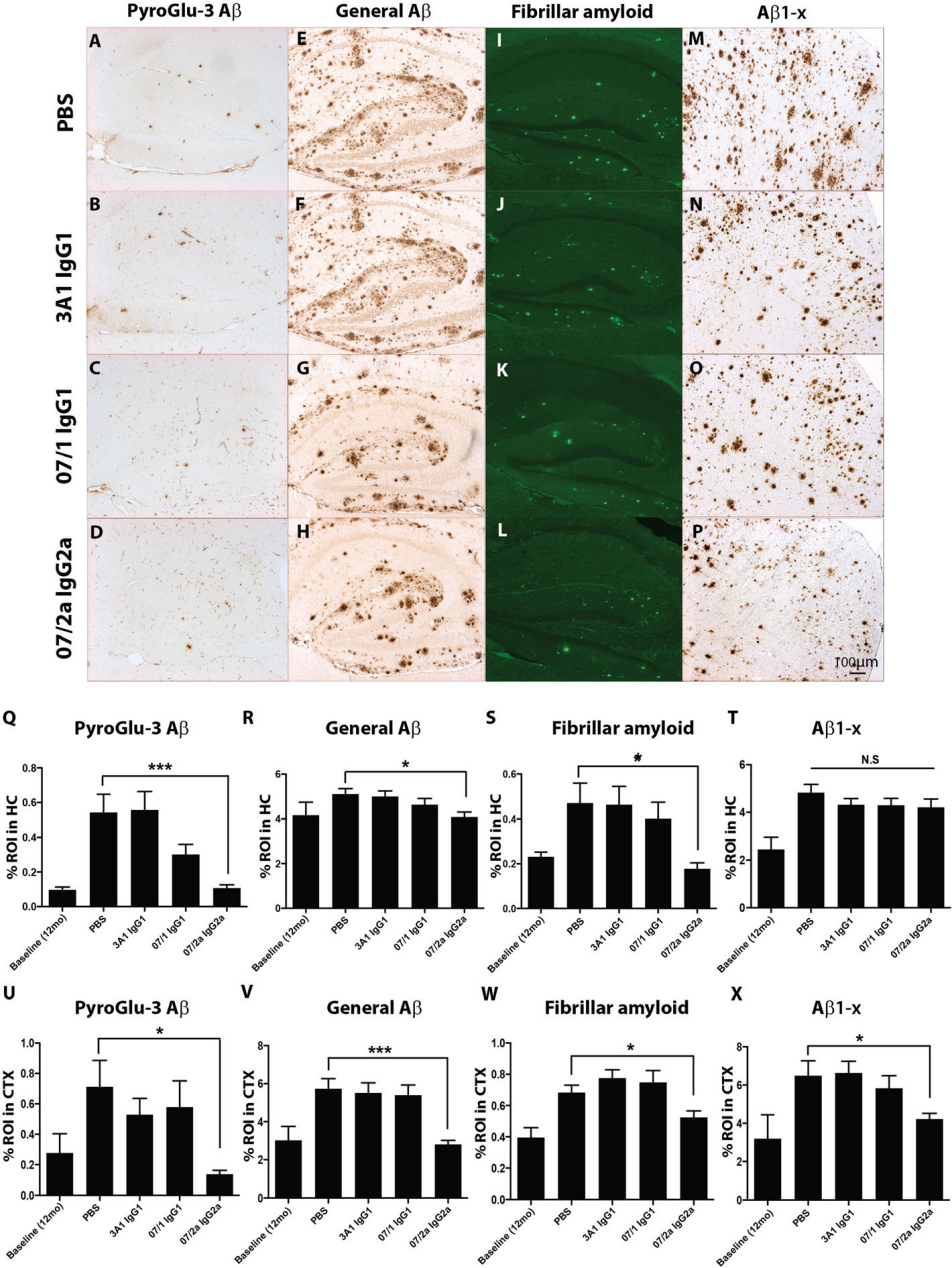
Lowering of multiple Aβ species in aged APP_SWE_/PS1ΔE9 Tg brains following 07/2a IgG2a mAb immunotherapy. Representative photomicrographs of hippocampus (HC) or cortex (CTX) from each treatment group of serial sections immunolabeled with a novel pGlu-3 Aβ IgG2b monoclonal antibody (**a**–**d**; HC), a general Aβ polyclonal antibody, R1282 (**e**–**h**; HC), stained with a marker for fibrillar amyloid, Thioflavin S (**i**–**l**; HC), and with a marker that specifically recognizes Aβ starting at Asp1, 82E1 (**m**–**p**; CTX). Quantitative image analysis on six stained sections at thee equidistant planes per marker demonstrated that there was a significant reduction of pGlu-3 Aβ (*p* < 0.001), general Aβ (*p* < 0.05), and fibrillar amyloid (*p* < 0.05) in the hippocampus of Tg mice immunized with 07/2a IgG2a mAb compared to Tg mice treated with PBS (**q**–**t**). Similarly, there was a significant reduction of pGlu-3 Aβ (*p* < 0.05), general Aβ, fibrillar amyloid, and Aβ_1−x_ (*p* < 0.05) in the cortex of the Tg mice immunized with 07/2a IgG2a mAb compared to Tg mice treated with PBS (**u**–**x**). *n* = 13–15 per group. All data, excluding baseline, are expressed as the mean ± SEM. ANOVA with Neuman-Keuls post test: ***p < 0.001 and *p < 0.05 versus Tg PBS

Antibody and PBS-injected mice were sacrificed 1 week following the final injection at 16 mo of age. Tissues were harvested and analyzed for neuropathological and biochemical changes in the CNS and periphery after immunotherapy. In order to assess the potential differences between 07/1 and 07/2a on Aβ clearance in vivo, we examined mouse brain sections using quantitative immunohistochemistry. Aβ plaque burden was assessed in the brains of the five APP_SWE_/PS1ΔE9 Tg mice sacrificed at ~ 12 mo of age as baseline controls. Immunostaining of fixed brain tissue sections with 07/2b (pGlu-3 Aβ IgG2b), R1282 (general Aβ), and 82E1 (Aβ1-x) showed immunoreactivity (IR) in the hippocampus (HC) and cortex (CTX) (Fig. 2a–h, m–p) in all APP_SWE_/PS1ΔE9 Tg mice. Thioflavin S-positive fibrillar amyloid plaques were also observed in the HC and CTX (Fig. 2i–l). Plaque burden increased from baseline (12 mo) to 16 mo of age.

Pyroglu-3 Aβ IR, measured by 07/2b mAb, to avoid cross-reactivity between the immunizing mAb (07/1 or 07/2a) and secondary antibody, was reduced dramatically by 80% in the HC (*p* < 0.001) and by 81% in the CTX (*p* < 0.05) of the 07/2a-treated Tg mice compared to PBS-injected mice (Fig. 2a–d, q, u). 07/2a-specific significant reductions were also observed for general Aβ deposition in the HC (*p* < 0.05) and CTX (*p* < 0.001) where R1282 IR was reduced by 20% and 51%, respectively, compared to Tg PBS-injected mice (Fig. 2e–h, r, v). Quantification of Aβ1-x measured by 82E1 IR showed no significant differences between the antibody treatment groups and PBS-injected mice in the HC (Fig. 2t). However, there was a 35% reduction (*p* < 0.05) of 82E1 IR in the CTX of 07/2a-treated mice compared to PBS-injected mice (Fig. 2m–p, x). Thioflavin S-positive fibrillar amyloid plaques were reduced by 62% (*p* < 0.05) in HC and 23% (*p* < 0.05) in CTX in 07/2a-treated Tg mice compared to PBS controls (Fig. 2i–l, s, w). There were no significant differences in Aβ load as analyzed by one-way ANOVA between PBS-injected Tg mice and 07/1 and 3A1 mAb-treated mice in either the HC or CTX.

**Fig. 3:**
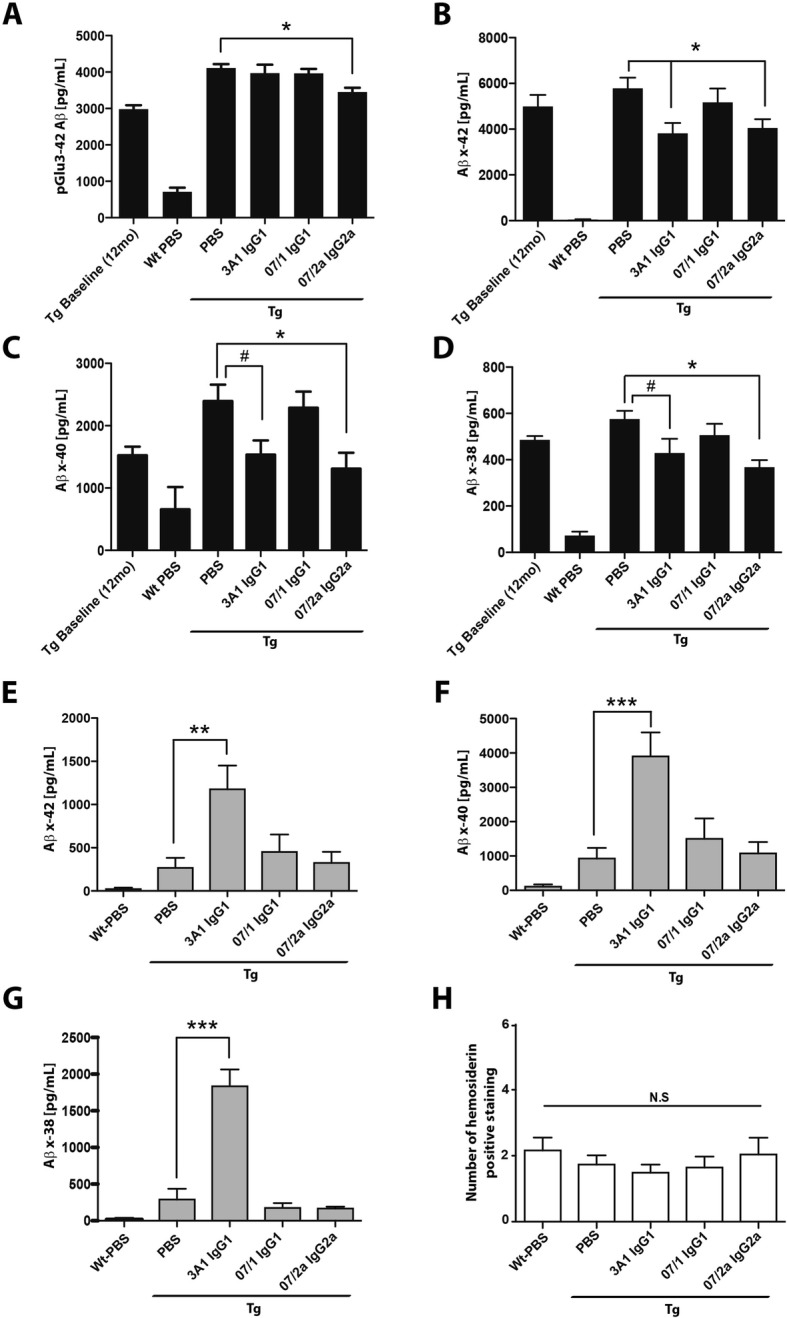
07/2a IgG2a and 3A1 IgG1 mAb’s lowered guanidine-HCl-extracted Aβ in aged APP_SWE_/PS1ΔE9 mice. Significant decreases in guanidine-HCl-extracted pGlu-3 Aβ (*p* < 0.05), Aβx-42 (*p* < 0.01), Aβx-40 (*p* < 0.05), and Aβx-38 (*p* < 0.05) were observed in Tg mice treated with 07/2a IgG2a mAb compared to PBS-treated Tg mice (**a**–**d**). Similarly, treatment with 3A1 mAb reduced guanidine-HCl-extracted Aβx-42 (*p* < 0.01), Aβx-40 (*p* < 0.05, *t*-test), and Aβx-38 (*p* < 0.05, *t*-test) in Tg mice compared to PBS controls (**b**–**d**). Treatment of the Tg mice with 3A1 IgG1 mAb resulted in a significant increase in plasma levels of Aβx-42 (0.01), Aβx-40 (*p* < 0.001), and Aβx-38 (*p* < 0.001) with no differences observed in Tg mice treated with anti-pGlu-3 Aβ mAbs, 07/1 IgG1 or 07/2a IgG2a, compared to PBS-treated Tg mice (**e**–**g**). No significant (N.S) differences in hemosiderin-positive staining were observed between antibody-treated Tg mice versus PBS-injected Tg mice or WT mice (**h**). *n* = 13–15 per group. All data, excluding baseline, are expressed as the mean ± SEM. ANOVA with Neuman-Keuls post test: ***p < 0.001, **p < 0.01, and *p < 0.05 versus Tg PBS. Student’s *t*-test: ^#^*p* < 0.05

Passive immunization against Aβ in mouse models has demonstrated an increase in cerebral microhemorrhages hypothesized to be due to movement of parenchymal plaques to the vasculature for clearance following antibody treatment [33, 34] or possibly because of direct binding of antibodies to existing cerebral amyloid angiopathy [35]. Therefore, we sought to quantify the occurrence of microhemorrhages in this study. Hemosiderin staining was performed for the detection of microhemorrhages across the entirety of three equidistant sagittal cross sections of the mouse brain by three independent investigators blinded to the treatment of each mouse. No significant differences were observed between antibody treatment groups and PBS controls (Fig. 3h) indicating that none of the antibodies increased the incidence of microhemorrhage.

### 07/2a treatment reduced guanidine-HCl-extracted Aβ levels in brain homogenates

Biochemical analysis of guanidine-HCl-extracted Aβ from whole brain (excluding cerebellum) homogenates demonstrated a significantly reduced concentration of pGlu-3Aβ in the insoluble fraction from 07/2a-treated Tg mice compared to Tg PBS control mice (*p* < 0.05) (Fig. 3a). The levels of insoluble Aβx-42 were significantly lower in the 07/2a- and 3A1-treated mice compared with Tg PBS-injected mice (*p* < 0.05) (Fig. 3b). In addition, 07/2a-treated mice had significantly lower Aβx-40 (*p* < 0.05) and Aβx-38 (*p* < 0.05) in the insoluble Aβ fraction relative to Tg PBS control mice (Fig. 3c, d). A trend for reduction in Aβx-40 and Aβx-42 was observed by 3A1-treated mice compared to PBS-injected Tg mice and was significant when compared to PBS controls by Student’s *t* test (*p* < 0.05) (Fig. 3c, d). There were no significant changes in soluble Aβx-40 and Aβx-42 levels in the T-PER extracted Aβ homogenates (data not shown) and the pGlu-3 Aβ and Aβx-38 levels in this fraction were below the detection limit for their respective Aβ ELISAs.

### 07/2a treatment did not alter plasma Aβ levels

Reduction of Aβ burden by altering the equilibrium between CNS and plasma Aβ, otherwise known as the “peripheral sink” hypothesis, has been demonstrated in previous immunotherapy studies as a mechanism of plaque reduction in the brain [4, 36]. To investigate if there were changes in peripheral Aβ levels in the antibody-treated Tg mice, terminal plasma was collected from all mice and Aβ was measured by an MSD immunoassay. Tg mice treated with 3A1 showed dramatic increases in Aβx-42 (*p* < 0.01), Aβx-40 (*p* < 0.001), and Aβx-38 (*p* < 0.001) levels compared with Tg PBS control mice (Fig. 3e–g). Interestingly, the pGlu-3 Aβ mAbs, 07/1 and 07/2a, showed no significant changes in Aβ levels compared to Tg PBS control mice suggesting less antibody binding in the periphery and, perhaps, an alternative mechanism of plaque removal. Similar to our prevention study [12], we did not see detectable levels of pGlu-3 Aβ in the plasma in these APP_SWE_/PS1ΔE9 Tg mice (data not shown). We also examined cytokine and chemokine levels in the plasma of the mice in this study to determine if the treatments had any pro- or anti-inflammatory effects; however, after measuring for changes in IFNγ, IL-2, IL-4, IL-5, IL-6, IL-10, IL-12p70, KC-GRO, and TNFα levels by MSD immunoplatform (Additional file 1: Figure S1), only KC-GRO (also known as CXCL1 chemokine) levels demonstrated a difference with 07/2a-treated mice showing a significant reduction (*p* < 0.05) compared to PBS-injected transgenic mice (Additional file 1: Figure S1H).

### Exogenous antibody concentrations were elevated in the CNS and periphery according to the immunizing antibody

The amount of 07/1, 07/2a, and 3A1 exogenous antibodies present at sacrifice in the CNS and plasma were measured by ELISA in the T-PER soluble brain homogenates (Additional file 1: Figure S2A-C) and plasma (Additional file 1: Figure S2D-F). We observed an increase in plasma pGlu-3 Aβ antibody levels in 07/1 (~ 52,000 ng/mL) and 07/2a-treated mice (~ 58,000 ng/mL) (Additional file 1: Figure S2E-F). No antibodies recognizing pGlu3–12 Aβ were detected in plasma from 3A1 or PBS-injected mice, supporting 07/1 and 3A1 mAb’s specificities (Additional file 1: Figure S2E-F). In contrast, circulating antibodies that recognized Aβ1–18 were present in 3A1-immunized mouse plasma (~ 82,000 ng/mL) (Additional file 1: Figure S2D) but not in either of the pGlu-3 Aβ immunization groups or PBS-injected Tg controls (Additional file 1: Figure S2D). In parallel, increases in exogenous antibody levels, albeit at lower concentrations, were observed in the T-PER soluble brain fractions from saline-perfused mice. This demonstrates that a fraction of the exogenous antibodies administered in the periphery were able to penetrate the CNS. In the brain, ~ 250,000 pg/mL antibodies that recognized pGlu3–12 Aβ from 07/1 immunized Tg mice and ~ 425,000 pg/mL antibodies from 07/2a immunized mice were detected (Additional file 1: Figure S2B-C). No pGlu3-Aβ-specific antibodies were detected in soluble brain homogenates from 3A1-immunized or PBS-injected mice (Additional file 1: Figure S2B-C). Although lower than the amount of Aβ1–18-recognizing antibodies that were detected in the plasma, soluble brain homogenates from 3A1-immunized mice had concentrations of 40,000 pg/mL antibodies (Additional file 1: Figure S2A). There was a very small amount of Aβ1-18-recognizing antibodies in brain in the 07/1-treated mice but no appreciable amounts from 07/2a- or PBS-injected Tg mice detected (Additional file 1: Figure S2A). We also carried out immunohistochemical analysis on brain sections with biotinylated anti-mouse IgG1 and anti-mouse IgG2a secondary antibodies alone to detect the immunizing antibodies in brain. Adjacent sections were stained with R1282 and pGlu-3 Aβ 07/2b antibodies to identify plaques. Biotinylated IgG1 secondary antibody detected a subset of cerebral plaques in 3A1-treated and 07/1-treated mice while biotinylated IgG2a secondary antibody recognized a subset of plaques in 07/2a-treated mice (Additional file 1: Figure S3).

### 07/2a treatment increased plaque-associated microgliosis in the hippocampus and cortex

**Fig. 4:**
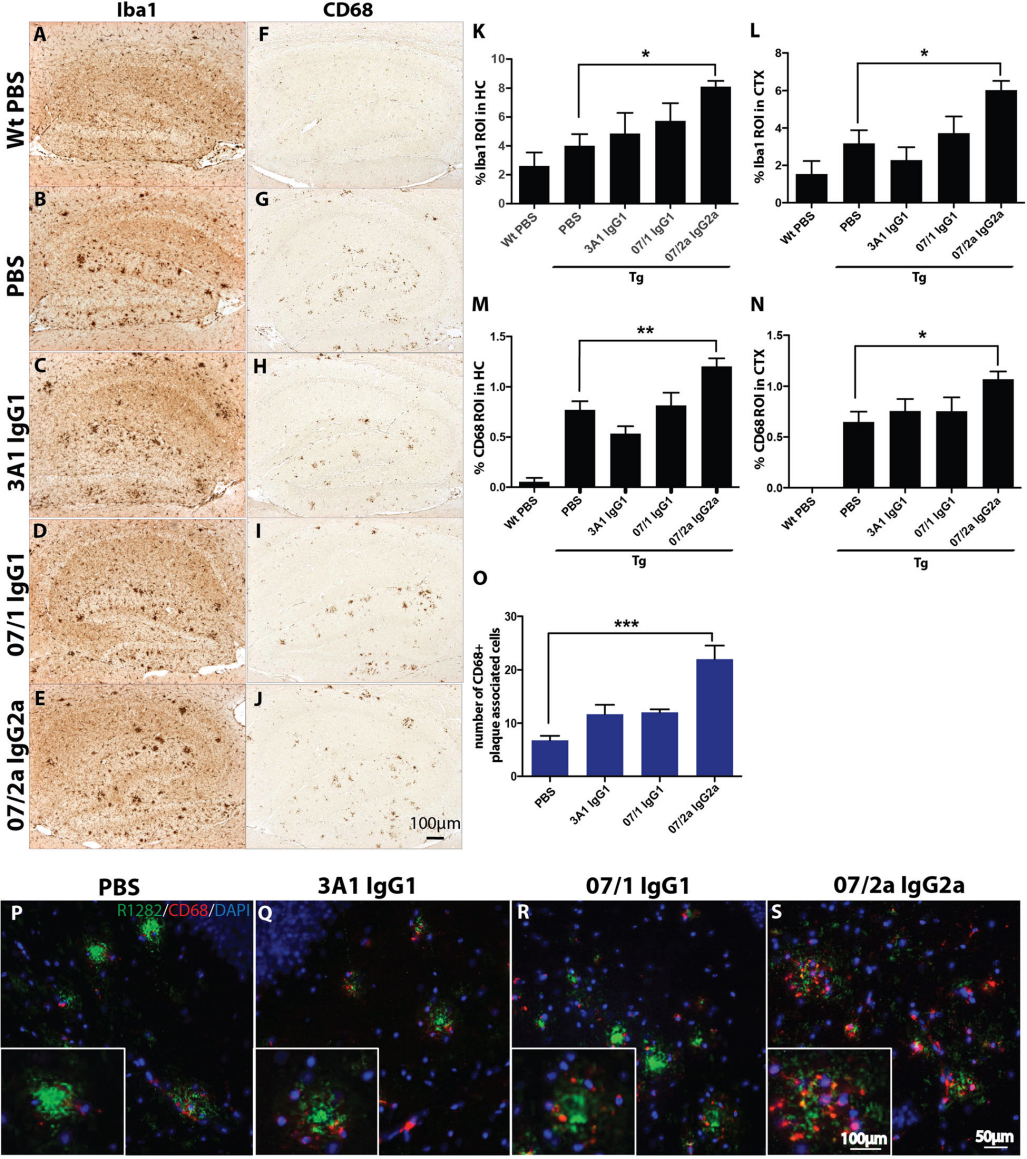
Immunohistochemical analysis of microglia and macrophage markers in the hippocampus and cortex of APP_SWE_/PS1ΔE9 Tg mice. Passive immunization, in aged APP_SWE_/PS1ΔE9 mice, resulted in a significant increase in plaque-associated microglia in mice treated with 07/2a IgG2a mAb. Representative photomicrographs of the hippocampus from each treatment group immunolabeled with microglial and macrophage marker, Iba1 (**a**–**e**), and activated microglial and macrophage marker, CD68 (**f**–**j**), showed an increased immunolabeling in immunized mice compared to PBS-treated Tg mice. Image analysis demonstrated that there was a significant increase in the percent area of Iba1-positive staining in the hippocampus (*p* < 0.05; **k**) and cortex (*p* < 0.05; **l**) of 07/2a IgG2a mAb-immunized mice compared to PBS-treated Tg mice. Analysis of the cortex demonstrated that there was a significant increase in CD68-positive immunolabeling in the hippocampus (*p* < 0.01; **m**) and cortex (*p* < 0.05; **n**) of 07/2a IgG2a mAb-immunized mice compared to PBS-treated Tg mice. A significant increase in plaque-associated CD68-positive cells was observed in Tg mice immunized with 07/2a IgG2a mAb (**o**, **s**). *n* = 13–15 per group. All data are expressed as the mean ± SEM. ANOVA with Neuman-Keuls post test: **p < 0.01 and *p < 0.05 versus Tg PBS

To evaluate if there were changes in glial accumulation in HC and CTX of these mice, IR with a marker for microglia and macrophage, Iba1, was quantified on three sections at equidistant planes. A significant increase in Iba1-positive staining was observed in the mice treated with 07/2a compared to PBS-injected Tg mice in both the HC (*p* < 0.05) and the CTX (*p* < 0.05) but not observed in the groups treated with 07/1 and 3A1 (Fig. 4a–e, k, l). We also measured IR in these treatment groups with a microglial and macrophage marker of phagocytosis, CD68. We observed from analysis of this phagocytic marker that 07/2a was the only mAb treatment to demonstrate a significant increase compared to PBS control Tg mice in the HC (Fig. 4m; *p* < 0.01) and CTX (Fig. 4n; *p* < 0.05). We carried out double immunofluorescence (IF) staining on the tissue with a general Aβ marker, R1282, and CD68 and measured, specifically in the hippocampus, the number of CD68-positive cells surrounding plaques. We found that 07/2a-treated Tg mice had a significant amount of CD68-positive, plaque-associated cells as semi-quantitatively measured compared to the PBS control mice (Fig. 4o, s).

### The IgG subclass of anti-pGlu3-antibodies affected Aβ phagocytosis ex vivo

**Fig. 5:**
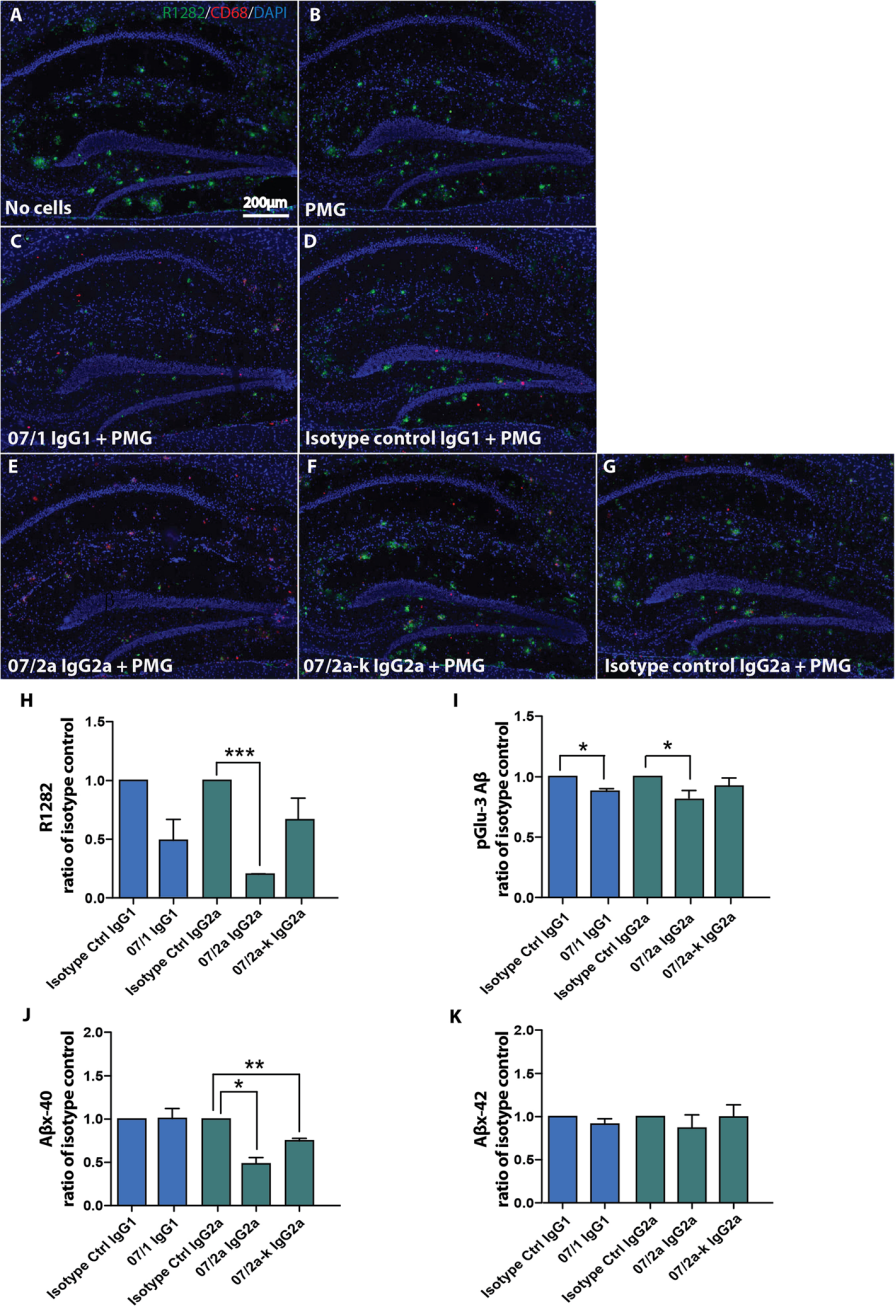
Ex vivo antibody-mediated phagocytosis assay demonstrates differences in plaque clearance based on antibody isotype. Unfixed, frozen plaque-rich tissue sections (20 μm) from 20-mo-old APP_SWE_/PS1ΔE9 Tg mice were pre-incubated with the following antibodies for 1 h: 07/1 IgG1, isotype control IgG1, 07/2a IgG2a, 07/2a-k IgG2a, or isotype control IgG2a. Following washing, the tissue was incubated with primary murine microglia (PMG) for 24 h and Aβ levels were determined. Representative photomicrographs of the hippocampus from each treatment group co-immunolabeled with a general Aβ marker, R1282, an activated microglial and macrophage marker, CD68 and a nucleic acid dye, DAPI (**a**-**g**) and showed decreased R1282 immunolabeling in the hippocampus of tissue pre-incubated with 07/2a IgG2a compared to isotype control (**a**–**h**). pGlu-3 Aβ, Aβx-40, and Aβx-42 levels were also determined by ELISA (**i**–**k**). Significant reductions in pGlu-3 Aβ were observed in tissue pre-incubated with 07/1 IgG1 and 07/2 IgG2a mAbs compared to their respective isotype controls (**i**). Tissue incubation with 07/2a IgG2a and 07/2a-k IgG2a resulted in decreased Aβx-40 levels compared to isotype control (**j**). There were no differences in Aβx-42 levels observed between groups (**k**). *n* = 3 per group. All data are expressed as the mean ± SEM. ANOVA with Neuman-Keuls post test: ***p < 0.001, **p < 0.01, and *p < 0.05 versus isotype control

In order to address the mechanism of plaque removal by 07/1, 07/2a, and 07/2a-k by potentially mediating microglial phagocytosis of Aβ, we assessed plaque clearance by microglia from unfixed, frozen brain tissue of aged APP_SWE_/PS1ΔE9 Tg mice in an ex vivo phagocytosis assay. Previous studies have demonstrated that Aβ antibody added to tissue sections, binds to plaques, and initiates plaque clearance through Fc-receptor-mediated phagocytosis [4, 5]. Prior to initiation of the assay, we first determined whether the pGlu-3-42 Aβ epitope was accessible in the unfixed frozen tissue from a 20-mo-old APP_SWE_/PS1ΔE9 mouse. Robust immunofluorescence detection with 07/1 mAb was observed in the cortex and hippocampus, with staining to a lesser extent observed in the cerebellum (Additional file 1: Figure S4A-C), suggesting sufficient pGlu-3-42 antigen was present to allow for Fc-receptor-mediated phagocytosis. Ex vivo phagocytosis assays were conducted, and Aβ plaque load/levels were measured by either immunofluorescent staining or ELISA, and the results were expressed as a ratio of the relevant isotype control. There were no significant differences in R1282 IR (data not shown) between tissue that had 24 h incubation with media alone (no cells) (Fig. 5a) and incubation with PMG (Fig. 5b), suggesting that without the addition of an Aβ antibody, phagocytosis is negligible. Similar R1282 IR levels were also seen in the tissue pre-incubated with IgG isotype control antibodies and PMG compared with PMG alone, eliminating non-specific Fc-receptor binding initiating clearance (Fig. 5d, g). Following 24 h incubation of the tissue with PMG, there was a significant reduction in R1282-positive staining present in the tissue pre-incubated with 07/2a mAb compared to incubation with IgG2a isotype control (*p* < 0.001) whereas exposure with the pGlu-3 Aβ IgG1 and 07/2a-k mAbs did not promote a significant reduction (Fig. 5a–h). Analysis of the guanidine-HCl insoluble Aβ homogenates by ELISA demonstrated that there was a significant decrease in pGlu-3 Aβ following incubation with the tissue with both 07/1 (*p* < 0.05) and 07/2a (*p* < 0.05) (Fig. 5i). Aβx-40 levels were only reduced with exogenous addition of the IgG2a antibodies, 07/2a (*p* < 0.05) and 07/2a-k (*p* < 0.01) (Fig. 5j), whereas there were no significant differences in Aβx-42 levels observed following incubation of any of the Aβ mAbs in the ex vivo phagocytosis assay (Fig. 5k). These results were mostly confirmed in another set of ex vivo phagocytosis assays using N9 cells, a murine immortalized microglial cell line (Additional file 1: Figure S5).

An accompanying in vitro assessment of the Fcγ receptor binding of 07/2a and 07/2a-k to isolated CD16, CD32, and CD64, i.e., the Fcγ receptor components, revealed no profound differences of the two antibodies (Table 1). In addition, binding affinities to pGlu-3 Aβ were similar between 07/1 (10.2 nM), 07/2a (9.8 nM), and 07/2a-k (8.08 nM).

**Table 1:** *K*_D_ values of antibody binding to Fcγ receptor subtypes based on ELISA using immobilized receptor

Fcγ receptor *K*_D_ (μg/ml)		
Receptor	07/2a IgG2a	07/2a-k IgG2a-k
CD16A	0.39	0.67
CD32	0.68	1.50
CD64	0.21	0.39

### 07/2a and 07/2a-k treatment of hAPP_SL_:hQC mice reduced Aβ

**Fig. 6:**
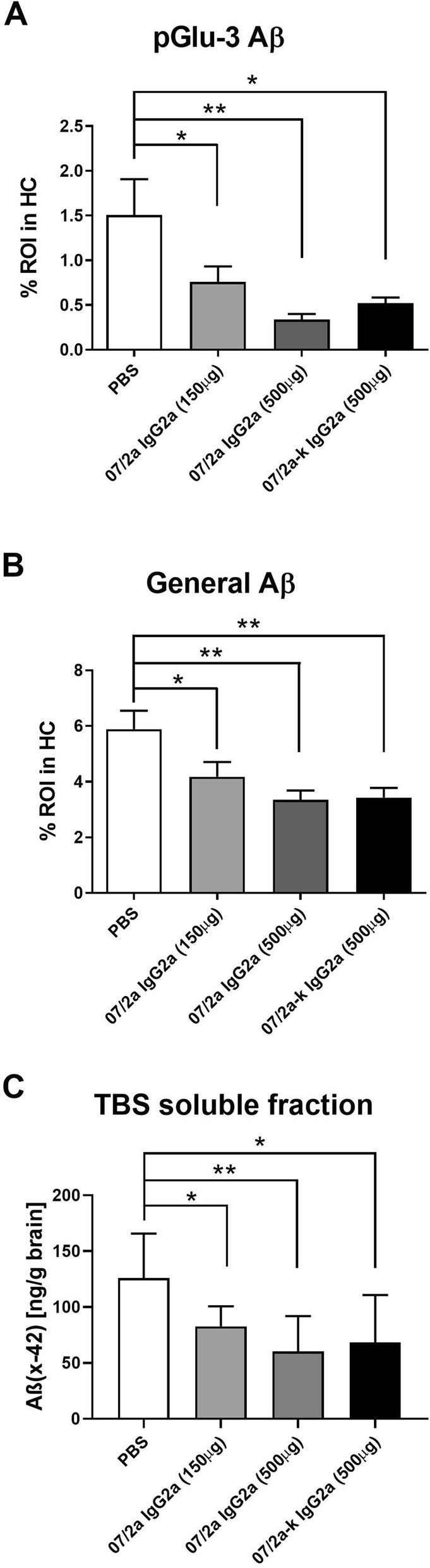
07/2a and 07/2a-k immunotherapy lowers Aβ in hAPP_SL_:hQC mice**.** Quantitative image analysis of the hippocampus on six stained sections at three equidistant planes per marker demonstrated that there was a significant reduction of pGlu-3 Aβ following 4 months of treatment with 07/2a at 150 μg (*p* < 0.05), 07/2a at 500 μg (*p* < 0.01), and 07/2a-k at 500 μg (*p* < 0.05) (**a**). General Aβ as measured by R1282 IHC demonstrated a reduction after dosing with 07/2a at 150 μg (*p* < 0.05), 07/2a at 500 μg (*p* < 0.01), and 07/2a-k at 500 μg (*p* < 0.01) (**b**). Aβ_x-42_ levels measured in the TBS soluble fraction were reduced in the brains of animals treated with 07/2a at 150 μg (*p* < 0.05), 07/2a at 500 μg (*p* < 0.01), and 07/2a-k at 500 μg (*p* < 0.05) (**c**). *n* = 7–8 mice per group. All data are expressed as the mean ± SEM. ANOVA with Bartlett’s post test: ***p < 0.001, **p < 0.01, and *p < 0.05 versus isotype control

We next compared two doses of the 07/2a antibody (150 and 500 μg) and one dose of 07/2a-k (500 μg) for their ability to clear Aβ in a plaque-rich AD model. Overall, Aβ clearance was similar between 07/2a-k, compared to that seen with 07/2a (Fig. 6). pGlu-3 Aβ (*p* < 0.05) and general Aβ (*p* < 0.01) immunoreactivity in the hippocampus showed significant reductions by 07/2a-k, similar to that of 07/2a-treated mice (Fig. 6a, b). The plaque-lowering potential of the CDC mutant antibody was further confirmed in the TBS soluble fraction isolated from the hemibrain of the treated mice and potentially containing toxic oligomers (Fig. 6c), although insoluble Aβ levels were not significantly altered. 07/2a IgG2a immunotherapy in these mice demonstrated a dose-dependent effect in Aβ reduction (Fig. 6a–c).

### Longitudinal PET imaging demonstrated treatment with pGlu-3 Aβ mAbs of different IgG isotypes resulted in distinct glial activation characteristics

Different Ig isotypes of antibody used in passive immunization studies can have various influences on microglial activation, which may result in different mechanisms of Aβ clearance. Thus, it is important to compare different Ig isotypes against the same target for their effect on clearing Aβ and inducing transient and/or chronic neuroinflammation. We performed microPET imaging using a second-generation TSPO (translocator protein, a marker for activated microglia) PET tracer, ^18^F-GE180, to visualize the differences in microglial activation prior (baseline, day 0) to and following i.p. passive immunization (days 3 and 30) with 3 anti-pyroGlu3 Aβ mAbs (07/1 IgG1, 07/2a IgG2a, and 07/2a-k, a CDC mutant version of 07/2a IgG2a to avoid complement activation), a general Aβ IgG1 mAb (3A1), or PBS in young (pre-plaque, 4-mo-old) or aged (plaque-rich, 14–16-mo-old) male APP_SWE_/PS1ΔE9 Tg mice.

**Fig. 7:**
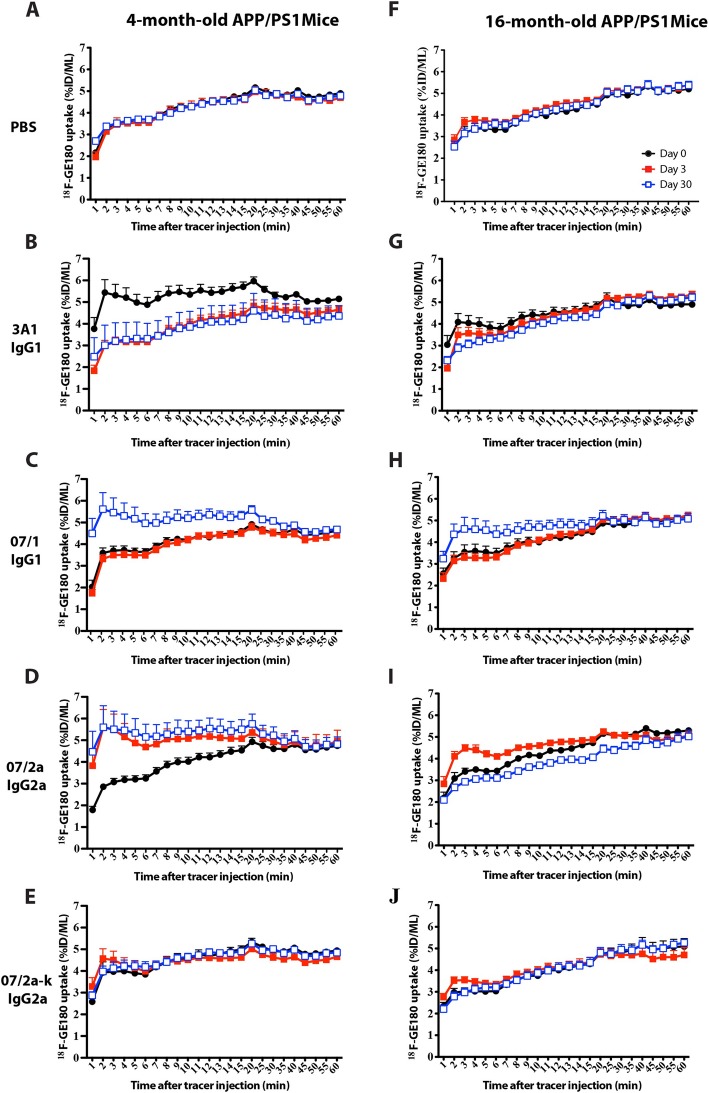
Uptake of ^18^F-GE180 tracer in mouse hippocampus at day 0, day 3, and day 30. One-hour microPET imaging was carried out using a novel TSPO radioligand, ^18^F-GE180, to visualize the microglia activation in mouse brain pre- and post-antibody immunization. The tracer uptake was quantitatively estimated as the percentage of injected dose per unit volume of hippocampus. **a**–**e** Time-activity curve (TAC) of hippocampal ^18^F-GE180 uptake in 4-mo-old mice with a single injection of 0.25 ml PBS (**a**) or 500 μg of antibody including 3A1-IgG1 (**b**), 07/1-IgG1 (**c**), 07/2a-IgG2a (**d**), or 07/2a-k (**e**). **e**–**h** TAC of hippocampal ^18^F-GE180 uptake in 16-mo-old mice with a single injection of 0.25 ml PBS (**f**) or 500 μg of antibody including 3A1-IgG1 (**g**), 07/1-IgG1 (**h**), 07/2a-IgG2a (**i**), or 07/2a-k IgG2a (**j**). *n* = 3–4 mice per treatment group per age. Two-way ANOVA with Bonferroni post hoc test was applied for statistical analysis

The time-activity curve (TAC) of ^18^F-GE180 uptake in the hippocampus was analyzed using CT/PET/MRI co-registration. There were no differences in hippocampal TAC of ^18^F-GE180 at day 0 (baseline), 3, or 30 in 4-mo-old and 16-mo-old APP_SWE_/PS1ΔE9 mice with PBS injection (Fig. 7a, f). Following a single injection of 07/1 mAb, a significant increase in hippocampal ^18^F-GE180 uptake was found at day 30 versus baseline in 4-mo-old (*F*_(1,8)_ = 5.62; *p* < 0.05) (Fig. 7c) and 16-mo-old (*F*_(1,8)_ = 10.54; *p* < 0.05) mice (Fig. 7h). Four-month-old APP/PS1dE9 mice injected with 07/2a antibody showed overt elevation in ^18^F-GE180 uptake at day 3 (*F*_(1,8)_ = 3.52; *p* = 0.097, trend) and day 30 (*F*_(1,8)_ = 5.62; *p* < 0.05) compared to baseline (Fig. 7d), while 16-mo-old APP/PS1dE9 mice treated with the same antibody showed a significant increase in hippocampal ^18^F-GE180 uptake at day 3 (*F*_(1,8)_ = 7.28; *p* < 0.05) but a decrease at day 30 (*F*_(1,8)_ = 15.84; *p* < 0.01) (Fig. 7i). No significant changes in hippocampal ^18^F-GE180 uptake were observed at day 3 or day 30 at either age in mice treated with 07/2a-k mAb (Fig. 7e, j). Although the 3A1-injected 4-mo-old APP/PS1dE9 mice showed a consistent reduction of ^18^F-GE180 hippocampal uptake at day 3 (*F*_(1,8)_ = 18.0; *p* < 0.01) and 30 versus baseline (*F*_(1,8)_ = 3.77; *p* = 0.088, trend), these mice had abnormally high baseline tracer uptake (Fig. 7b). Similar results were observed in the whole brain TAC of ^18^F-GE180 uptake (Additional file 1: Figure S6).

## Discussion

The presence of pGlu-3 Aβ in human brains exclusively under pathological conditions, in addition to its increased stability and propensity to aggregate, makes this peptide an attractive therapeutic target [14, 18, 20]. Compelling evidence now suggests that the N-terminal modification induces formation of toxic structures such as oligomers and membrane pores [17, 19, 22, 37, 38]. Therefore, anti-pGlu-3 Aβ treatment strategies by inhibition of glutaminyl cyclase or immunotherapy are currently in development [32, 39]. The targeting of pGlu-3 Aβ by passive immunotherapy differs from other Aβ antibody treatments in various aspects: (i) pGlu-3 Aβ is a highly pathologic and neurotoxic species, (ii) the targeting of the N-terminal Aβ modification efficiently rules out binding to parent molecules (e.g., APP and non-modified beta CTF), preventing potential side effects, and (iii) pGlu-3 Aβ is undetectable in plasma; thus, there is no “peripheral capture” of the drug expected in the circulation [12].

Accordingly, our nonclinical prevention studies have demonstrated a lowering of plaque burden when targeting pGlu-3 Aβ early in young Tg mice by immunotherapy [12, 32, 40]. The aim of this study was to investigate the effect of passive immunization of murine anti-pGlu-3 Aβ mAbs of different IgG isotypes, in a therapeutic paradigm, on cognition and plaque burden in aged, plaque-rich APP_SWE_/PS1ΔE9 Tg mice and, thus, to provide avenues for the humanization of the drug molecule. A pilot study previously conducted by our lab demonstrated an attenuation of Aβ plaque deposition in aged APP_SWE_/PS1ΔE9 Tg mice following peripheral administration of 07/1 mAb after the onset of cerebral Aβ deposition [24]; however, this small study did not examine the effects of administration of an IgG2a-specific pGlu-3 Aβ mAb nor the effects of both antibody isotypes on cognition. Therefore, these were amongst the main objectives of the present study.

In our present therapeutic study, pathological examination of chronic treatment effects of anti-pGlu3 Aβ IgG1 and IgG2a mAbs demonstrated reduced brain amyloid plaque burden and brain pGlu-3 Aβ, Aβx-42, Aβx-40, and Aβx-38 peptide levels. These results, in part, corroborated findings published by Eli Lilly [32], describing a reduction in guanidine-HCl extracted Aβ1–42 in the hippocampus and cortex of PDAPP mice treated for 3 months with an anti-pGlu-3 Aβ antibody, mE8 IgG2a. However, the Lilly study did not show any differences measured by histopathological analysis. In our current study, 07/2a treatment not only demonstrated a decrease of cerebral pGlu-3 Aβ plaque deposition but also other forms of Aβ in the brain suggesting that by reducing pGlu-3 Aβ, it removed the seeds for further plaque deposition, a mechanism by which pGlu-3 Aβ has been reported to trigger AD pathogenesis [19]. Interestingly, we observed a significant increase in the amount of positive microglial/macrophage staining in the hippocampus and cortex of the 07/2a-treated mice compared to control PBS mice, suggesting that the Aβ reduction observed in this immunotherapy study may be a result of a microglial-mediated mechanism of removal of amyloid deposits in the Tg mouse brain. Early studies have shown that microglia not only accumulate around Aβ plaques in AD, but are also able to contribute to their clearance [41, 42]. Mutations on microglial receptors that play a role in phagocytosis have been associated with an increased risk of developing AD [43–46] making this mechanism, or lack thereof, a strong contending explanation for the accumulation of Aβ observed in AD. IgG antibodies can act as opsonins, which in the context of this study, bind to general Aβ or pGlu-3 Aβ, to tag them for phagocytosis through recognition of the Fc portion of the antibody by the Fc receptors on phagocytes [5, 47]. We were able to measure a significant increase of CD68-positive staining, which is a marker for phagocytic microglia and macrophage, surrounding the plaques in the hippocampus of the anti-Aβ mAb-treated mice compared to the PBS-treated mice, with 07/2a-treated mice showing the most robust plaque-lowering, further indicating this mechanism of clearance for this antibody.

Further confirmation of microglial activation by 07/2a IgG2a antibody was demonstrated by a significant increase in hippocampal and whole brain ^18^F-GE180 uptake, a second-generation radioligand of TSPO (a marker for activated microglia), as measured by in vivo microPET imaging in the 4-mo- and 16-mo-old mice 3 days following a single injection of 07/2a. Interestingly, microglial activation remained at high levels 30 days after 07/2a injection as compared to its baseline (day 0) in 4-mo-old mice that barely have pGlu-3 Aβ pathologies; however, microglial activation was decreased at 30 days after 07/2a injection in 16-mo-old APP/PS1dE9 mice that were loaded with pGlu-3 Aβ pathologies, implying that immunization of IgG2a isotype of an anti-pGlu-3 Aβ mAb can effectively and rapidly evoke microglial reactivity in mouse brain within 72 h, while chronic clearance of pGlu-3 Aβ by even a single-dose injection of 07/2a can result in a reduction in brain inflammation.

Interestingly, the strong effect of 07/2a to elicit phagocytosis, Aβ and pGlu-Aβ clearance, and microglial activation also resulted in a cognitive improvement of the mice. We assessed learning and memory in the mice using the water T-maze behavioral paradigm. Although the treatment did not achieve a full rescue of spatial learning and memory, it clearly suggested that only the 07/2a mAb provoked substantial cognitive benefits to these aged mice. In addition, 07/2a had the highest penetration into the brain followed by 07/1, both of which were much higher than 3A1. Taken together, these results collectively suggest that a strong effector function of an anti-pGlu-3 antibody and good brain exposure are required to attenuate pathological changes and behavioral abnormalities in aged transgenic mice. Considering the effect on microglial activation and inflammation, it is important that we did not observe an incidence of microhemorrhages. Immunotherapy in aged APP transgenic mice can pose the risk of creating microbleeds [33, 35], which has been a fairly accurate predictor of what has occurred in the clinics with some treatments [48, 49]. Although our mice were 12 months of age at the start of immunization and 16 months at study completion, it may be possible that the incidence of microbleeds would be higher in even older APP/PS1 mice, as demonstrated by Li et al. when comparing microhemorrhage in middle-aged APP/PSI mice and old APP mice following a 3-month immunotherapy regimen [50].

Although none of the treatments showed an increased risk of microbleeds, we observed differences between the antibodies in the potential to reduce Aβ burden in the brain and increase plasma Aβ levels. A number of mechanisms for Aβ clearance have been suggested such as the breakdown of amyloid deposits [51, 52], removal of cerebral Aβ through “pulling” Aβ from brain to plasma, termed “peripheral sink” [4, 36], and Aβ plaque removal through microglial-mediated phagocytosis [53, 54]. In our study, the increased levels of Aβ species measured in the plasma of saline-perfused Tg mice treated with our positive control, 3A1, compared with the Tg PBS-injected mice, are consistent with a “peripheral sink” mechanism of CNS plaque removal. In contrast, Aβ levels were similar across the groups treated with the anti-pGlu-3 Aβ mAbs and PBS controls suggesting that the anti-pGlu-3 Aβ mAbs work through an alternative mechanism to remove Aβ from the brain—a conclusion which is also supported by the increased CD68 staining. The significant contribution of the peripheral sink mechanism in case of 3A1 might also explain the low level of Aβ reduction in our ex vivo phagocytosis assay.

While a different mode of action might contribute to the differential results obtained with 3A1, 07/1, 07/2a, and 07/2a-k, also the brain penetration of the drug may be of importance. Penetrance of peripherally administered antibodies into the brain remains a challenge of immunotherapy treatment with typically only approximately 0.1% of antibodies are thought to cross the blood brain barrier (BBB) into the CNS [55]. Our immunohistochemical data with only secondary antibodies demonstrated target engagement of 3A1, 07/1, and 07/2a with plaques in the brain. In the 07/2a-treated mice, we saw ~ 1.7× increase in concentration of pGlu-3 Aβ IgG2a in brain homogenates compared with levels of pGlu-3Aβ IgG1 in the mice treated with 07/1 mAb. 07/2a mAb may have better accessibility into the brain compared with 07/1 resulting in better treatment outcomes. In agreement with other studies, we did not detect pGlu-3 Aβ in the plasma of any of the mice in this study [12, 56, 57]; therefore, 07/1 and 07/2a mAbs could not have been saturated in the periphery, unlike the 3A1 mAb, and as such provided better ability to work through a CNS-mediated process. Accordingly, 3A1 concentration in the brain homogenate was much lower compared to 07/1. The half-lives of murine IgG isotypes, IgG1, and IgG2a have been shown to be similar (6–8 days) [58].

Taken together, our results suggest that a strong effector function and stimulation of phagocytosis is required for pGlu3-Aβ antibodies under consideration for a therapeutic trial. With amyloid immunotherapy trials being dampened by reports of ARIA, it is becoming more important to consider effector function. It was thought that Fc effector function could be a prominent contributor to the vascular side effects observed in clinical trials; therefore, AC immune developed crenezumab (licensed by Genentech), which is a humanized anti-Aβ mAb that binds multiple forms of Aβ and is designed as an IgG4 isotype to limit inflammatory cytokine release while keeping phagocytic function [59]. However, disappointing results from trials were observed and further analyses using a murine-generated IgG2a equivalent of crenezumab provided evidence that in the absence of side effects, the ability of the antibody to engage plaques is also important to ensure a therapeutic effect [60]. This would suggest that effector function alone is not solely important when developing a treatment and requires intense consideration for every antibody showing different specificity. In addition to the eminent role of the effector function, our comparison with 3A1, an antibody detecting the intact Aβ N-terminus which does not recognize APP, clearly suggests that the specificity of the antibody epitope influences the mode of action and, possibly, the distribution to the brain.

Although aducanumab showed clinical benefit in a Phase III clinical trial in patients with mild cognitive impairment or early stages of AD, and donanemab cleared plaques in Phase 1a/b trials, both antibodies induced vasogenic edema visualized by MRI as ARIA-E. Therefore, from a translational perspective, a strong effector function in addition to a good Aβ target in the absence of inducing inflammation is important for a therapeutic effect in patients. In order to meet these criteria, we introduced a CDC point mutation to 07/2a. Our data from ex vivo phagocytosis assays, the interaction profile with Fcγ receptors in vitro and the treatment of double transgenic mice suggest that the effector function to elicit phagocytosis was largely conserved. Importantly, the brain inflammatory changes due to treatment with the CDC-mutant antibody were significantly reduced compared with the IgG2a antibody as analyzed by ^18^F-GE180 uptake. Thus, using an antibody like this may be useful for tackling ARIA observed in human clinical trials. Further nonclinical immunotherapy studies are currently underway to investigate this antibody further and to examine such in vivo effects.

## Conclusions

Overall, we found that reduction of cognitive deficits and cerebral plaque load were associated with strong effector function of a murine anti-pGlu-3 Aβ mAb in aged, plaque-rich APP/PS1dE9 mice. Antibody engineering to reduce CDC-mediated complement binding facilitated plaque clearance and led to the reduction of neuroinflammation in vivo. Hence, our results suggest that targeting pathogenic pGlu-3 Aβ and tailoring the effector function of humanized antibodies may be useful for modulating the phagocytic clearance and attenuating the neuroinflammatory response. The murine anti-pGlu3 Aβ IgG2a (07/2a) and IgG2a-k (07/2a-k) mAbs have been humanized and de-immunized for clinical development and their characterization is under review for publication.

## Supplementary information

Additional file 1: Supplemental online material

## Abbreviations

AD: Alzheimer’s disease
ADCC: Antibody-dependent cellular toxicity
ARIA: Amyloid-related imaging abnormalities
Aβ: Amyloid-beta
BBB: Blood brain barrier
CDC: Complement-dependent cytotoxicity
CFC: Contextual fear conditioning
CNS: Central nervous system
CTX: Cortex
DS: Down syndrome
HC: Hippocampus
i.p: Intraperitoneal
Ig: Immunoglobulin
IR: Immunoreactivity
PBS: Phosphate buffered saline
pGlu-3: Pyroglutamate-3
PMG: Primary microglia
Tg: Transgenic
TSPO: Translocator protein
Wt: Wildtype
