# Supplementary material for: Effector function of anti-pyroglutamate-3 Aβ antibodies affects cognitive benefit, glial activation and amyloid clearance in Alzheimer’s-like mice
Source: Alzheimers Res Ther. 2020 Jan 13;12:12. doi: 10.1186/s13195-019-0579-8 (PMC6958628; doi:10.1186/s13195-019-0579-8)
Supplement: Supplementary file 1 — Supplemental online material [file 13195_2019_579_MOESM1_ESM.docx]

Effector function of anti-pyroglutamate-3 Aβ antibodies affects cognitive improvement, glial activation and amyloid clearance

Supplemental Online Material

**S1. Supplemental Materials and Methods**

S1.1 Euthanasia and tissue preparation

The brain was extracted and divided sagittally into two hemispheres. One hemisphere was snap frozen for biochemical analyses and the other hemisphere was drop-fixed in 4% paraformaldehyde (PFA) (Electron Microscopy Series) for 24 h at 4°C, cryoprotected in graded sucrose solutions at 4°C and embedded in OCT compound (Tissue Tek).

S1.2 Histology and image analysis

Neuropathological analysis of Aβ plaque burden and associated gliosis were carried out with the following antibodies: 07/2b (1:500), a pGlu-3 Aβ IgG2b mAb (Probiodrug AG), R1282 (1:1000), a general Aβ rabbit polyclonal antibody (pAb) (gift D. Selkoe, Boston, MA); 82E1 (1:100), a mouse monoclonal antibody recognizing Aβ1-x (IBL); Iba1 (1:500), a rabbit pAb that detects resting and activating microglia and macrophages (Wako Chemicals); and CD68 (1:200), rat mAb that detects activated microglia and macrophages with a phagocytic phenotype (AbD Serotec). Sections were air-dried for 20 minutes and washed with TBS for 2 x 5 mins. Immunohistochemistry was carried out using the Elite ABC Kit (Vector Laboratories). Fibrillar amyloid was stained for using 1% Thioflavin S (Sigma-Aldrich) and microhemorrhages were visualized by staining with hemosiderin (2% potassium ferrocyanide in 2% hydrochloric acid) as previously described [21].

Quantitative image analysis of the percent area of immunoreactivity (IR) and Thioflavin S staining was carried out using Bioquant image analysis software (Nashville, TN) on six sections per mouse at three equidistant planes for hippocampus and frontal cortex for 07/2b, R1282, Thioflavin S and 82E1. The percent area of immunoreactivity for Iba1 and CD68 IR was quantified in the hippocampus and frontal cortex for three sections at three equidistant planes. The threshold of detection was held constant for each marker. Microhemorrhages were measured on two adjacent sections at three equidistant planes by counting the number of hemosiderin positive staining per entire sagittal section. The imager was blinded to the identity of the treatment of each animal.

S1.3 Brain homogenization and Aβ cytokine ELISA of immunotherapy homogenates

APP_SWE_/PS1ΔE9 mouse brain tissue without cerebellum was homogenized in tissue protein extraction reagent (T-PER) (Pierce) containing protease inhibitor cocktail (Roche), phosphatase inhibitor cocktails 2 and 3 (Sigma-Aldrich) using 120 Vac overhead stirrer (Wheaton), sonicated and centrifuged at 100,000 X g for 1 hr at 4°C. The supernatant was stored at -80°C and insoluble Aβ peptides were extracted from the pellet following re-homogenization with 5M Guanidine-hydrochloric acid (HCl) pH 8 in 50mM Tris containing protease inhibitor cocktail (Complete Mini, Roche), phosphatase inhibitor cocktails 2 and 3 (Sigma-Aldrich). The homogenates were incubated at 4°C for 3 hrs prior to centrifugation at 100,000 X g for 1 hr at 4°C. The solution was aliquoted and stored at -80°C until use.

Brain tissue of APPxhQC transgenic mice was homogenized in TBS (20 mM Tris, 137 mM NaCl, pH 7,6; 2 volumes of buffer per brain weight, Dounce homogenizer) containing protease inhibitor cocktail (Complete Mini, Roche) and 0.1 mM 4-benzenesulfonyl fluoride hydrochloride was sonicated and centrifuged at 75,500 x g for 1 hour at 4°C. The supernatant was stored at –80°C and Aβ peptides were sequentially extracted with 2.5 ml 2% SDS in distilled water (SDS fraction), and 0.5 ml 70% formic acid (formic acid fraction). The Formic acid extract was neutralized by addition of 3.5 M Tris solution and diluted to a final volume of 10 ml using ELISA blocking buffer (Pierce, Cat.No. 37571).

Aβx-38, Aβx-40, Aβx-42 (MesoScale Discovery) and pGlu-3-42 Aβ (IBL) were measured in the brain homogenates and terminal plasma samples by ELISA following the manufacturer’s instructions. Cytokine levels (IFNγ, IL-2, IL-4, IL-5, IL-6, IL-10, IL-12p70 and KC-GRO) were measured in the terminal plasma sample using a multiplex immunoassay platform (MesoScale Discovery) following manufacturer’s instructions. Antibody concentration in the brain homogenates and terminal plasma samples, were measured as described [12].

S1.4 Antibody concentration measurements in plasma and brain

Concentrations of the exogenous antibodies, 3A1, 07/1 and 07/2a, in the brain homogenates (whole brain excluding cerebellum) and the terminal plasma samples (1 week post-final injection) were measured were measured as follows. Streptavidin coated 96-well plates (Pierce) were washed 3 times with wash solution containing 25 mM Tris, 150 mM NaCl, 0.1% bovine serum albumin, and 0.05% Tween 20 at p.H 7.2 and subsequently coated with 30 ng of biotinylated Aβ1-18 peptide or 20 ng biotinylated pGlu3-12 Aβ (Probiodrug AG), for 2 hrs shaking at room temperature. The plates were then blocked with Pierce Protein-Free blocking buffer for 1 hr shaking at room temperature. The plates were washed 3 times and antibody standards and brain or plasma samples were added to the plates and incubated at 4°C for 2 hr. Following another additional 3 washes, the plates were incubated with 100 ng/mL anti-mouse IgG1 HRP or anti-mouse IgG2a HRP for 1 hr shaking at 4°C. The plates were washed 3 times and SureBlue TMB substrate solution (KPL) was added to each well for 30 minutes at room temperature in the dark. The detection reaction was stopped by adding 100 μL of 1.2 N H_2_SO_4_ and optical density values were measured at 450 nm using a Benchmark Microplate reader (Bio-Rad.).

S1.5 *Ex vivo* Phagocytosis assay

Following incubation with or without antibodies for 1 hr at 37°C in 5% CO_2_: 07/1 IgG1, 07/2a IgG2a, 07/2a-k IgG2a, isotype control IgG1 or isotype control IgG2a at optimized concentrations of 10 μg/ml for PMG cell assay or 15 μg/ml for N9 cell assay, the media was removed and the tissue was washed with PBS prior to the addition of PMG (3 X 10^5^) or N9 (1 X 10^5^) cells for 24 hr at 37°C in 5% CO_2_. Twenty-four hours after cell incubation, the media was collected (for cytokine ELISA) and the tissue was washed with PBS. Four wells per condition were homogenized in 5.2 M guanidine buffer (0.5 M guanidine in 50mM Tris-HCl at pH 8, 0.05% Tween20, 0.25% and protease inhibitor cocktail (Complete Mini, Roche)) (300 μl per well) as described by ^[24]^ and left at room temperature for 30 minutes prior to mechanical homogenization. The samples were further homogenized on an end-to-end rotator for 3 minutes at 4°C prior to quantification by ELISA. One well per condition was fixed in 4% PFA and prepared for immunoflouresence with R1282 (1:100) and CD68 (1:200).

S1.6 In vivo PET imaging with ^18^F-GE180 PET tracer and imaging analysis.

Mice were anesthetized with 3% isoflurane (Baxter Medical) and medical grade oxygen at a rate of 1 L/min. A 5-min CT scan was done first. The mice then received the same dose per gram of body weight, 1.75µCi/g ^18^F-GE180 tracer solution by tail vein injection, followed by a 0.1 mL saline flush. Dynamic PET imaging for each mouse was performed for 1h using a small animal PET/CT scanner (eXplore Vista; GE Healthcare). The spatial resolution of the PET scanner was ~1.6 mm at the center of the field-of-view. The data were acquired in 3D mode at the energy window of 250 –700 keV, which yields 4% count sensitivity. For imaging analysis, the PET data were reconstructed into 2D sinograms (frames: 1 min x 15, 5 min x 9) using Fourier rebinning and the conventional ordered-subsets expectation-maximization algorithm with 16 subsets and 2 iterations. Random and scattered coincidence events were corrected during the image reconstruction. The volume of interest (VOI) was placed on the whole brain by co-registration with CT images using MMWSK-V imaging analysis software and on hippocampus (HC) using AMIDE imaging analysis software with reference to the MRI template. The tracer uptake in each VOI was estimated as the percentage of injection dose per unit volume (%ID/ml). Statistical analysis was performed across the entire 60 min scanning interval.

**S2. Supplemental Analysis Results and Figures**


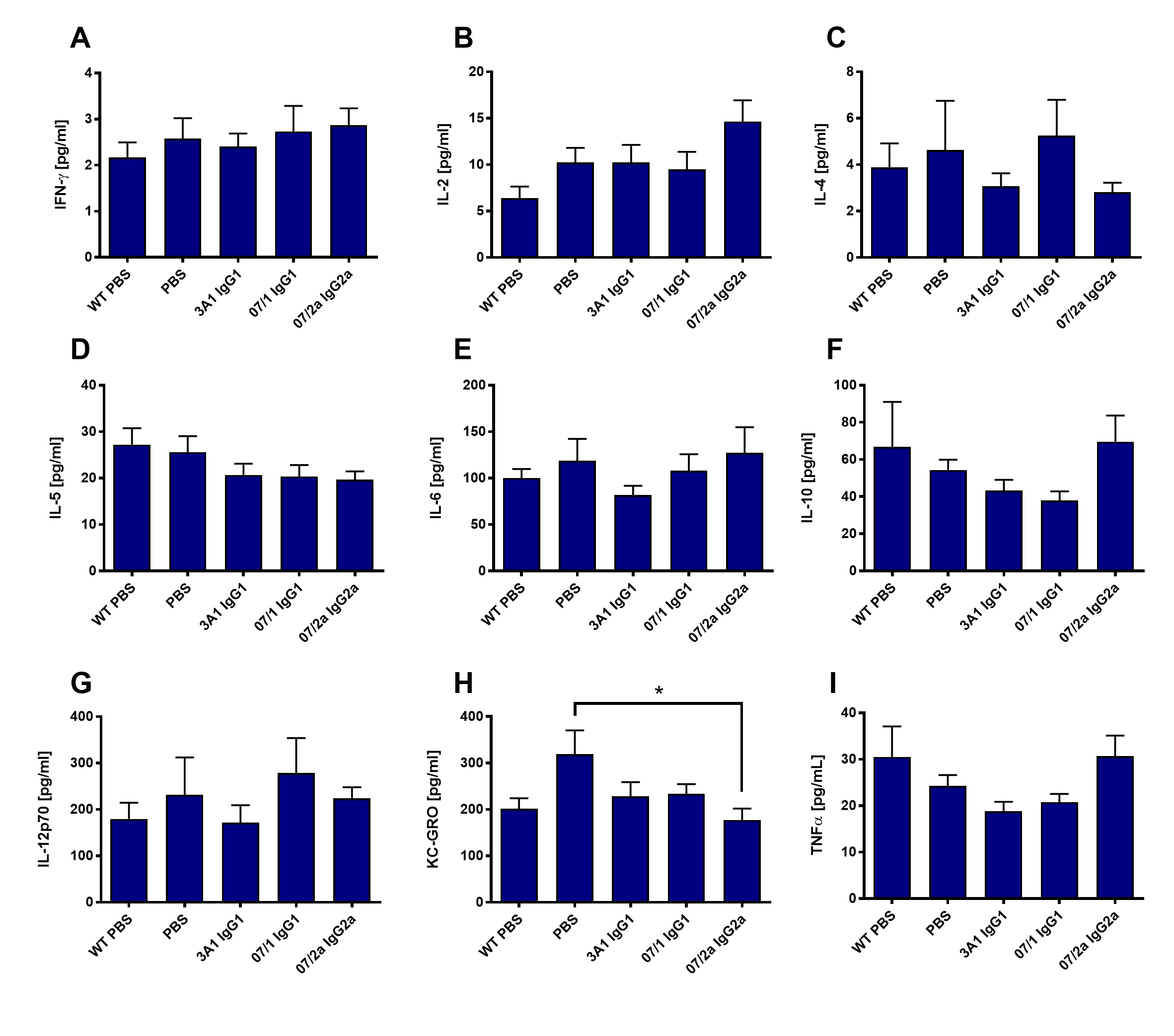


**Figure S1.** Plasma cytokine and chemokine levels. An MSD platform was used to determine IFNγ (A), IL-2 (B), IL-4 (C), IL-5 (D), IL-6 (E), IL-10 (F), IL-12p70 (G), KC-GRO (H), and TNFα levels in plasma from PBS, 3A1 IgG1, 07/1 IgG1 or 07/2a IgG2a treated mice. KC-GRO in plasma was significantly reduced in 07/2a IgG2a treated (versus PBS) animals (H). n=3 per group. All data are expressed as the mean ± SEM. ANOVA with Neuman-Keuls posthoc test: *P<0.05 versus PBS.


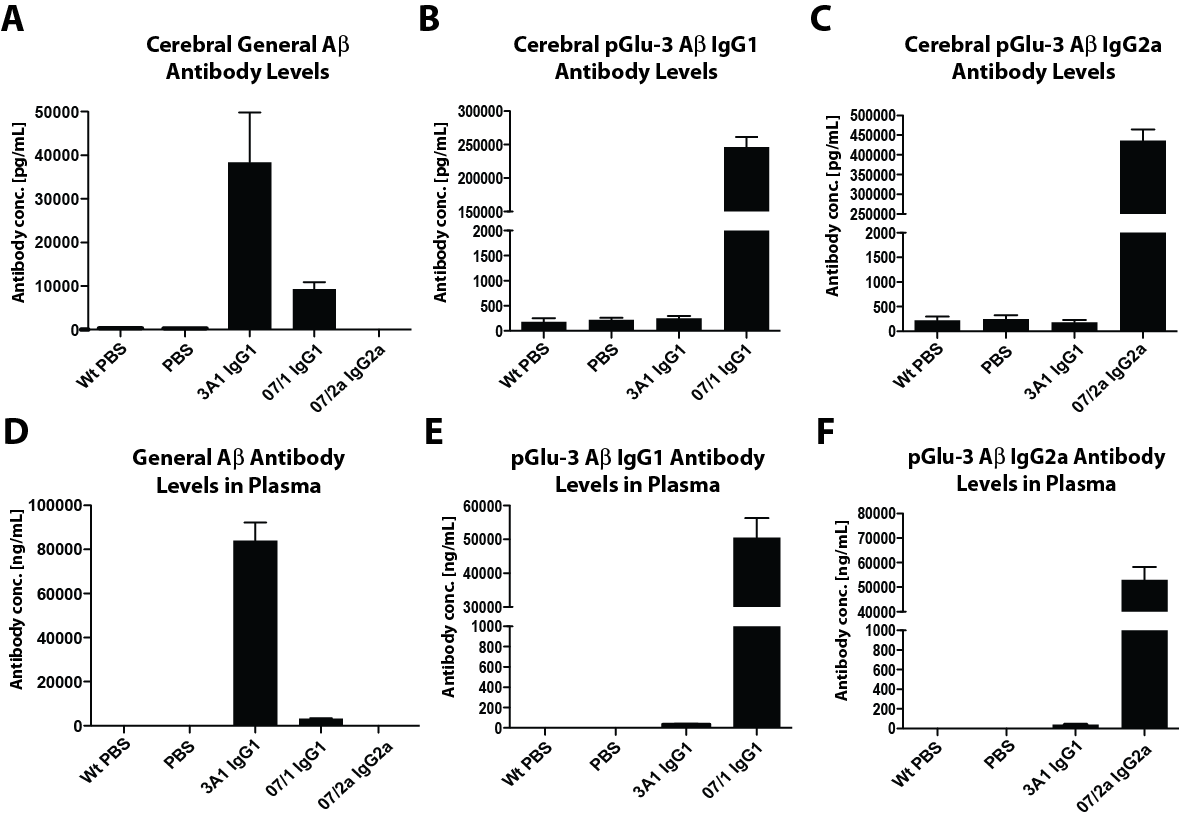


**Figure S2.** Antibody concentration ELISAs demonstrate levels of the i.p. injected antibodies in the brain (A-C) and plasma (D-F). Cerebral IgG1 antibodies recognizing Aβ1-18 were highest in 3A1 treated mice (A). Cerebral IgG1 antibodies recognizing pGlu-3 Aβ were highest in 07/1 treated mice (B), whilst IgG2a antibodies recognizing pGlu-3 Aβ were highest in 07/2a treated mice (C). Only mice chronically treated with 3A1 antibody had plasma levels of antibodies recognizing Aβ1-18 (D), whereas pGlu-3 Aβ IgG1 and IgG2a antibodies were only detected in plasma of 07/1 treated (E) and 07/2a treated (F) mice, respectively. n=10-13 per group.


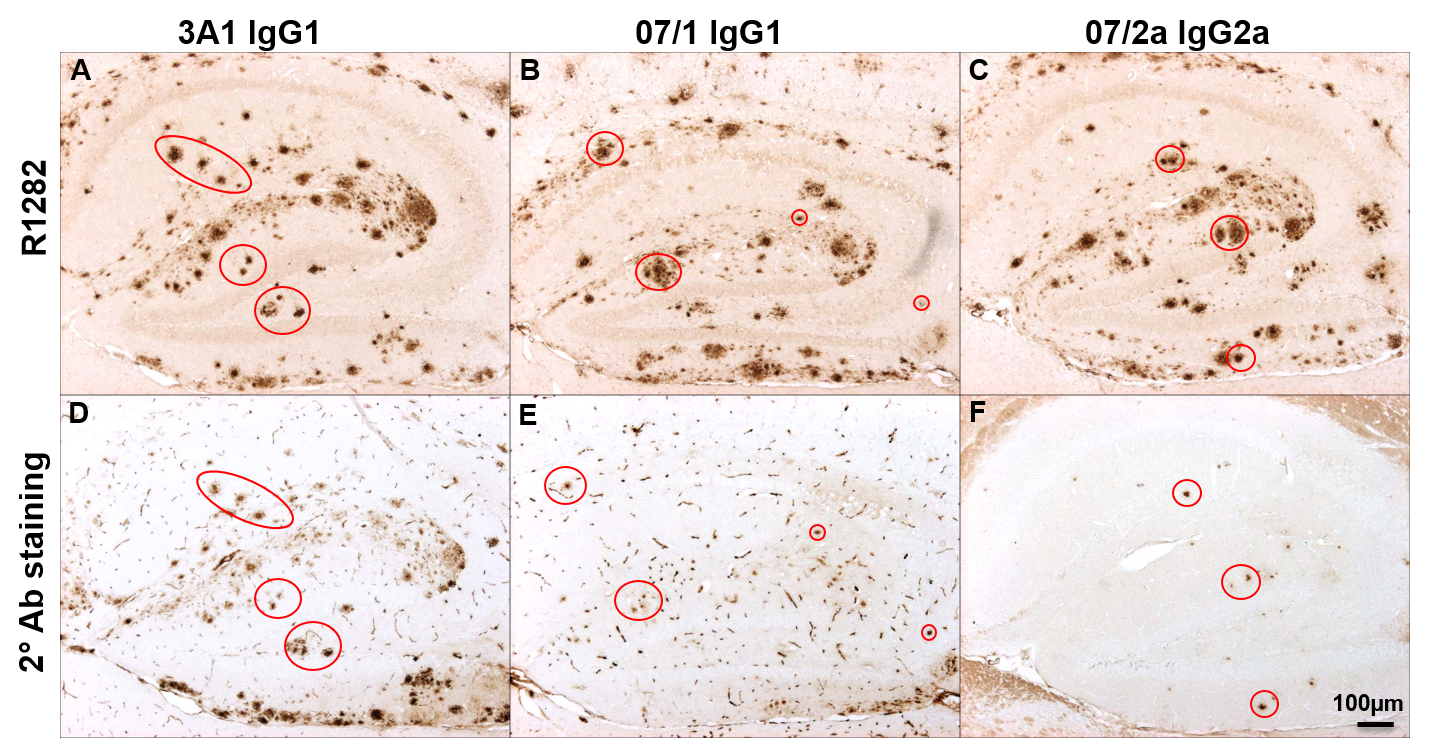


**Figure S3.** DAB staining of tissue using IgG1 and IgG2a secondary antibodies alone. Representative microphotographs of hippocampus from antibody treated animals show immunoreactivity in tissue probed with secondary antibody alone (D, E and F) in the vicinity of R1282-positive Aβ staining in an adjacent section (A, B and C).


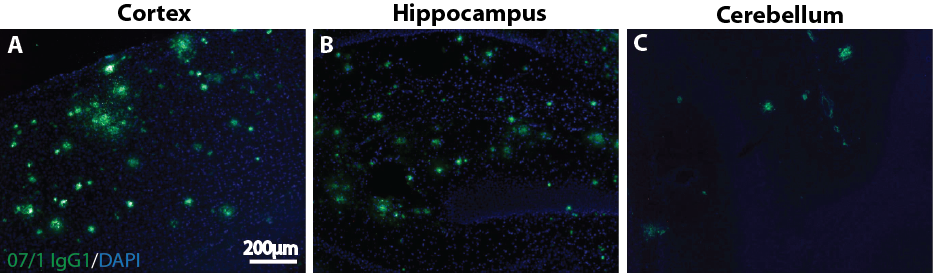


**Figure S4.** Immunoflourescence labeling with an anti-pyroglutamate-3 Aβ mAb in brain of a 20 mo-old APP_SWE_/PS1ΔE9 Tg mouse used in the *ex vivo* phagocytosis assays shows availability of epitope in sections. Unfixed, frozen plaque-rich brain tissue sections (20μm) from 20 mo old APP_SWE_/PS1ΔE9 Tg mice were immunoflourescently stained with the 07/1 IgG1 mAb. Photomicrographs show an abundance of positive staining in the cortex (A) and hippocampus (B) and positive staining to a lesser extent in the cerebellum (C).


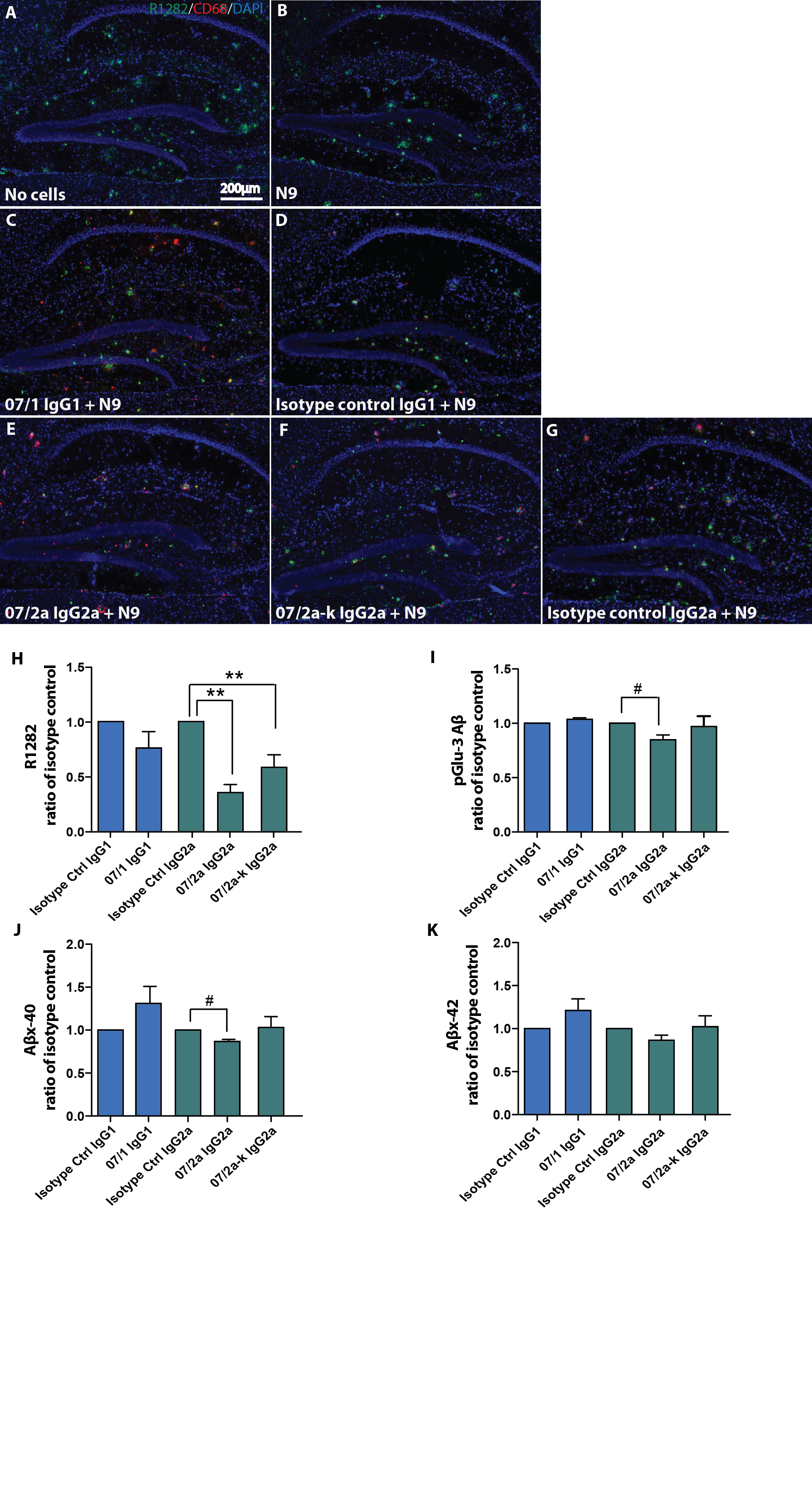


**Figure S5.** *Ex vivo* antibody mediated phagocytosis assay demonstrates differences in plaque clearance based on antibody isotype. Unfixed, frozen plaque-rich brain tissue sections (20μm) from 20 mo-old APP_SWE_/PS1ΔE9 Tg mice were pre-incubated with the following antibodies for 1hr: 07/1 IgG1, isotype control IgG1, 07/2a IgG2a, 07/2a-k, or isotype control IgG2a. Following washing, the tissue was incubated with N9 cells for 24 hr and Aβ levels were determined. Representative photomicrographs of hippocampus from each treatment group co-immunolabeled with a general Aβ marker, R1282, an activated microglial and macrophage marker, CD68 and a nucleic acid dye, DAPI (A-G) showed decreased R1282 immunolabeling in hippocampus of tissue pre-incubated with 07/2a and 07/2a-k compared to isotype control (E-H). pGlu-3 Aβ, Aβx-40 and Aβx-42 levels were also determined by ELISA (I-K). Modest but significant reduction in pGlu-3 Aβ was observed in tissue pre-incubated with 07/2 IgG2a mAb compared to its respective isotype control (I). Tissue incubation with 07/2a mAb resulted in decreased Aβx-40 levels compared to isotype control (J) and there were no differences in Aβx-42 levels observed between groups (K). n=3 per group. All data are expressed as the mean ± SEM. ANOVA with Neuman-Keuls posthoc test: **P<0.01 versus isotype control. Student’s t test: ^#^P<0.05 versus isotype control.

**
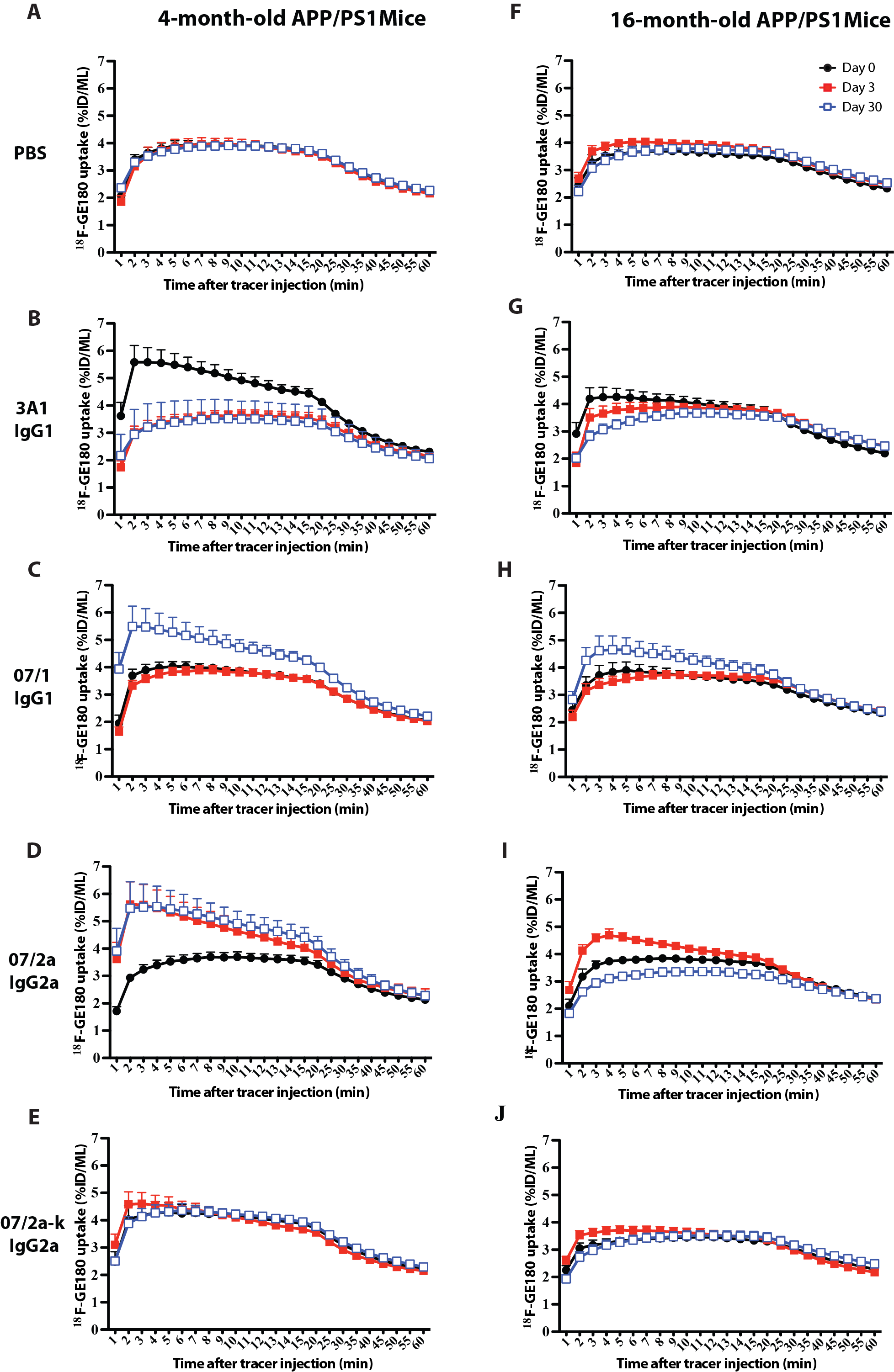
**

**Figure S6.** Uptake of ^18^F-GE180 tracer in whole brain of mouse at day 0 (baseline, pre-immunization), day 3 (post-immunization) and day 30 (post-immunization). One-hour microPET imaging was carried out using a novel TSPO (a marker for activated microglia) radioligand, ^18^F-GE180, to visualize the microglia activation in mouse brain pre- and post-antibody immunization.  A-D, Tracer-activity curve (TAC) of whole brain ^18^F-GE180 uptake in 4 mo-old mice with a single injection of 0.25 ml PBS (A) or 500 ug of antibody including 3A1 (B), 07/1 (C), 07/2a (D) or 07/2a-k (E). F-J, TAC of whole brain ^18^F-GE180 uptake in 16 mo-old mice with a single injection of 0.25 ml PBS (F) or 500 ug of antibody including 3A1 (G), 07/1 (H), 07/2a (I) or 07/2a-k (J). n=3-4 mice per treatment group per age. Two-way ANOVA with Bonferroni post-hoc test was applied for statistical analysis.
